# Optimal closed-loop deep brain stimulation using multiple independently controlled contacts

**DOI:** 10.1371/journal.pcbi.1009281

**Published:** 2021-08-06

**Authors:** Gihan Weerasinghe, Benoit Duchet, Christian Bick, Rafal Bogacz

**Affiliations:** 1 MRC Brain Network Dynamics Unit, Nuffield Department of Clinical Neurosciences, University of Oxford, Oxford, United Kingdom; 2 Department of Mathematics, Vrije Universiteit Amsterdam, Amsterdam, The Netherlands; 3 Systems and Network Neuroscience, Amsterdam Neuroscience, Amsterdam, The Netherlands; 4 Mathematical Institute, University of Oxford, Oxford, United Kingdom; 5 Department of Mathematics, University of Exeter, Exeter, United Kingdom; Université Paris Descartes, Centre National de la Recherche Scientifique, FRANCE

## Abstract

Deep brain stimulation (DBS) is a well-established treatment option for a variety of neurological disorders, including Parkinson’s disease and essential tremor. The symptoms of these disorders are known to be associated with pathological synchronous neural activity in the basal ganglia and thalamus. It is hypothesised that DBS acts to desynchronise this activity, leading to an overall reduction in symptoms. Electrodes with multiple independently controllable contacts are a recent development in DBS technology which have the potential to target one or more pathological regions with greater precision, reducing side effects and potentially increasing both the efficacy and efficiency of the treatment. The increased complexity of these systems, however, motivates the need to understand the effects of DBS when applied to multiple regions or neural populations within the brain. On the basis of a theoretical model, our paper addresses the question of how to best apply DBS to multiple neural populations to maximally desynchronise brain activity. Central to this are analytical expressions, which we derive, that predict how the symptom severity should change when stimulation is applied. Using these expressions, we construct a closed-loop DBS strategy describing how stimulation should be delivered to individual contacts using the phases and amplitudes of feedback signals. We simulate our method and compare it against two others found in the literature: coordinated reset and phase-locked stimulation. We also investigate the conditions for which our strategy is expected to yield the most benefit.

## Author summary

In this paper we use computer models of brain tissue to derive an optimal control algorithm for a recently developed new generation of deep brain stimulation (DBS) devices. DBS is a treatment for a variety of neurological disorders including Parkinson’s disease, essential tremor, depression and pain. There is a growing amount of evidence to suggest that delivering stimulation according to feedback from patients, or closed-loop, has the potential to improve the efficacy, efficiency and side effects of the treatment. An important recent development in DBS technology are electrodes with multiple independently controllable contacts and this paper is a theoretical study into the effects of using this new technology. On the basis of a theoretical model, we devise a closed-loop strategy and address the question of how to best apply DBS across multiple contacts to maximally desynchronise neural populations. We demonstrate using numerical simulation that, for the systems we consider, our methods are more effective than two well-known alternatives, namely phase-locked stimulation and coordinated reset. We also predict that the benefits of using multiple contacts should depend strongly on the intrinsic neuronal response. The insights from this work should lead to a better understanding of how to implement and optimise closed-loop multi-contact DBS systems which in turn should lead to more effective and efficient DBS treatments.

## Introduction

Deep brain stimulation (DBS) is an effective treatment for advanced Parkinson’s disease (PD) and essential tremor (ET) which involves delivering stimulation through electrodes implanted deep into the brain. Regions thought to be implicated in the disease are targeted in the treatment, which in the case of PD is typically the subthalamic nucleus (STN) and for ET the ventral intermediate nucleus (VIM) of the thalamus. PD is a common movement disorder caused by the death of dopaminergic neurons in the substantia nigra. Primarily, symptoms manifest as slowness of movement (bradykinesia), muscle stiffness (rigidity) and tremor. ET is purportedly the most common movement disorder, affecting just under 1% of the world population [1, 2] with the main symptom being involuntary shaking most commonly in the upper limbs [3]. Despite its prevalence, the pathophysiology of ET remains elusive, although the cortex, thalamus and cerebellum are all thought to be involved in the disease [2]. Symptoms of these disorders are thought to be due to overly synchronous activity within neural populations. For PD patients, higher power in the beta frequency range (13-30Hz) of the local field potential (LFP) measured in the STN has been shown to correlate with motor impairment [4] while thalamic activity in ET patients is strongly correlated with tremor measured using the wrist flexor EMG [5]. It is thought that DBS acts to desynchronise this pathological activity leading to a reduction in the symptom severity.

A typical DBS system consists of a lead, an implantable pulse generator (IPG) and a unit to be operated by the patient. The DBS lead terminates with an electrode, which is typically divided into multiple contacts. Post surgery, clinicians manually tune the various parameters of stimulation, such as the frequency, amplitude and pulse width, in an attempt to achieve optimal therapeutic benefit. Stimulation is then provided constantly, or ‘open-loop’, according to these parameters. The choice of stimulation frequency in particular is known to be crucial for efficacy with high frequency (HF) DBS (120-180 Hz) being found to be effective for both PD and ET patients [6, 7].

Despite the effectiveness of conventional HF DBS in treating PD and ET, it is believed that improvements to the efficiency and efficacy can be achieved by using more elaborate stimulation patterns informed by mathematical models. In particular, the link between neural synchrony and symptom severity have led to a number of theoretical studies into effective strategies for desynchronising systems of coupled phase oscillators [8–11]. Amongst these is coordinated reset (CR) neuromodulation, which is an open-loop DBS strategy where brief HF pulse trains are applied through different contacts of a stimulation electrode [8, 12–14]. In practice, CR has been shown to yield both acute and long-lasting benefits in parkinsonian monkeys and PD patients [12, 15].

Closed-loop stimulation and IPGs with multiple independent current sources are promising new advances in DBS technology. Closed-loop stimulation is a new development in DBS methods which aims to deliver stimulation on the basis of feedback from a patient. There is a growing body of evidence [4, 16–18] suggesting that closed-loop stimulation has the potential to offer improvements in terms of efficacy, efficiency and reduction in side effects. IPGs with multiple independent current sources are the ‘cutting-edge’ of DBS technology which, unlike their single current source counterparts, allow for current to be delivered independently to each contact. This gives increased control and flexibility over the shape of the electric fields delivered through the electrodes, allowing for more precise targeting of pathological regions and the possibility of delivering more complex potential fields over space, in addition to allowing for the possibility of recording activity from different regions. The use of multiple independently controllable contacts (which we will now simply refer to as multi-contact DBS), however, naturally leads to increased complexity, as many more stimulation strategies are now possible. This has created the need to better understand how applying DBS through multiple contacts can affect the treatment.

For closed-loop DBS, the choice, use and accuracy of feedback signals play a crucial role in determining the efficacy of the method. In the literature, both the LFP and tremor have been used as feedback signals with studies showing that the effects of DBS to be dependent on both the phase and amplitude of the oscillations at the time of stimulation [4, 17, 19, 20]. In adaptive DBS, high frequency stimulation is applied only when the amplitude of oscillations exceeds a certain threshold [4]. In phase-locked DBS stimulation is applied according to the instantaneous phase of the oscillations, which for ET patients corresponds to stimulation at roughly the tremor frequency (typically ∼ 5 Hz) [17]. The combined approach of adaptive and phase-locked stimulation has also been investigated in simulation [18].

### Summary of key results

**Fig 1:**
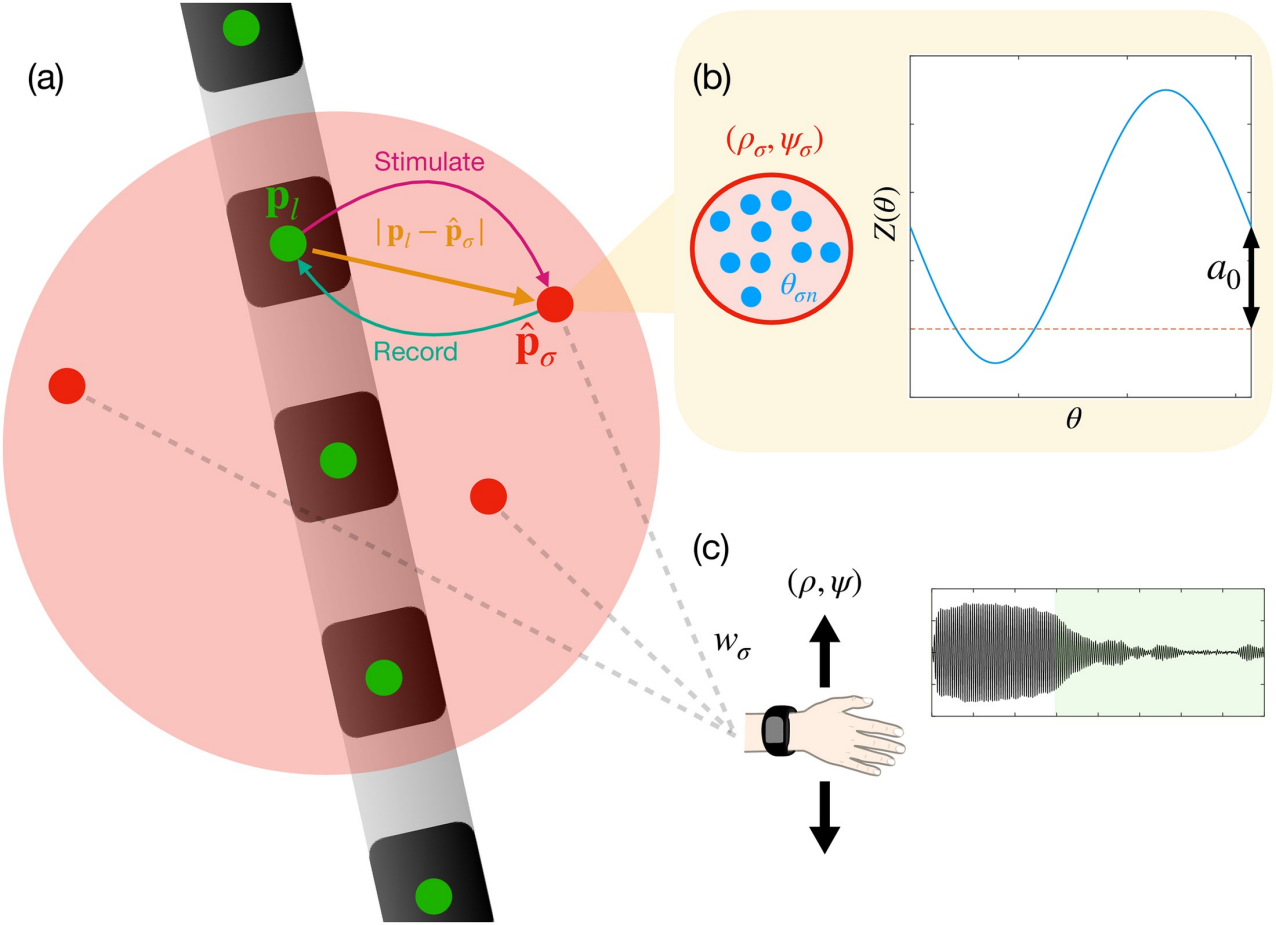
A model for closed-loop multi-contact DBS. (a) A multi-contact electrode is implanted into the VIM of the thalamus. Each contact (shown as green circles) delivers stimulation to and records from multiple coupled neural populations (shown as red circles), according to the geometry of the system. (b) We model each population as consisting of coupled neural units, shown as blue circles. Each unit is associated with a phase *θ*_*σn*_, which reflects where the neurons are in their firing cycle. The units of each population respond to stimulation according to a phase response curve *Z*(*θ*). The zeroth harmonic *a*_0_ (or the vertical shift) of this function plays an important role in determining efficacy. Each population is associated with a local amplitude *ρ*_*σ*_ and a local phase *ψ*_*σ*_, which are due to the collective activity of all units within the population. (c) The summed activity of multiple populations gives rise to symptoms, such as tremor in ET. The activities are summed according to a set of weights {*w*_*σ*_} and the resulting signal has an amplitude *ρ* and phase *ψ*. The amplitude *ρ* is correlated with the severity of tremor. When DBS with ACD is used (green region), the result is a reduction in *ρ* and thus the tremor severity. The effects are dependent on the positioning, measurement, and stimulation through multiple contacts.

In this work we propose a closed-loop DBS strategy designed for systems with multiple independently controllable contacts to optimally suppress disease-related symptoms by decreasing network synchrony; we refer to this strategy as adaptive coordinated desynchronisation (ACD). Our strategy builds on ideas introduced in earlier work for single contact systems [11]. ACD is derived on the basis of a model where multiple populations of neural units collectively give rise to a symptom related signal. The goal of ACD is to determine how DBS should be provided through multiple contacts in order to maximally desynchronise these units. The methods we present can be applied in different ways, either using multiple electrodes or single electrodes with multiple contacts. We therefore use the terms ‘electrode’ and ‘contact’ synonymously throughout. A summary of our model is illustrated in Fig 1. Key findings of our work are as follows:

1. We show that the effects of DBS for a multi-population Kuramoto system are dependent on the global (or collective) phase of the system and the local phase and amplitude which are specific to each population.
2. We show the effects of DBS can be decomposed into a sum of both global and local quantities.
3. We predict the utility of closed-loop multi-contact DBS to be strongly dependent on the zeroth harmonic of the phase response curve for a neural unit.
4. We predict the utility of closed-loop multi-contact DBS to be dependent on geometric factors relating to the electrode-population system and the extent to which the populations are synchronised.

## Models

To provide a foundation for the theory of multi-contact DBS, we first review in this section how stimulation with a single electrode acts on a single population of oscillators. Here we follow our previous paper [11], which may be referred to for a more detailed derivation of the results presented in this section. A list of frequently used notation is provided in Table 1.

**Table 1:** List of frequently used symbols together with their description.

Parameter	Description
*N*	Number of units
*S*	Number of populations
*L*	Number of electrodes
*θ*	Phase of oscillator
*ψ*	Phase of population
*ρ*	Synchrony of population
*r*	Complex order parameter
*k*	Coupling constant
*f*	Neural signal
*Z*	Unit phase response curve (uPRC)
*a*	Cosine Fourier coefficient of uPRC
*b*	Sine Fourier coefficient of uPRC
*ω*_0_	Centre of natural frequency distribution
*γ*	Width of natural frequency distribution
*w*	Population weight
Γ	Local amplitude response
*V*	Voltage at electrode
$\hat{V}$	Voltage at population
**p**	Electrode position
$\overset{´}{\text{p}}$	Neural compartment position
$\hat{\text{p}}$	Population position
*I*	Current at electrode
$\overset{´}{I}$	Current at neural compartment
$\hat{I}$	Current at population
**D**	Activity to voltage at electrode transformation matrix
$\tilde{\text{D}}$	Electrode to voltage at population transformation matrix
*d*	Element of **D**
$\tilde{d}$	Element of $\tilde{\text{D}}$
$\tilde{\sigma }$	Noise amplitude
*η*	Configuration parameter
Δ*θ*_max_	Maximum perturbation to single oscillator
*k*_diag_	Diagonal of coupling constant matrix
*s*_*a*_	Standard deviation of the zeroth harmonic of the uPRC

### The Kuramoto model

Modelling the effects of DBS generally poses a challenge since the brain networks involved in disorders such as ET (cortico-thalamic circuit) and PD (cortico-basal-ganglia circuit) are complex and it is still debated from which parts of these circuits the pathological oscillations originate [21, 22]. The task can be made more tractable by considering a simple phenomenological model which does not attempt to explicitly describe the underlying circuits, but rather focuses on general mechanisms leading to the synchronization of neural units [11]. We use the term ‘neural unit’ (or unit) to either mean regular-spiking neurons, bursting neurons or micro-circuits. The Kuramoto model [23, 24] describes the dynamics of these units as a system of homogeneously coupled oscillators, whose phases evolve according to a set of underlying differential equations. Such models are particularly attractive due to their simplicity and explicit dependence on phase, which makes them convenient for describing the effects of phase-locked stimulation [11].

We define the state of *N* neural units to be given by the set of oscillators {*θ*_1_(*t*), *θ*_2_(*t*), *θ*_3_(*t*)…*θ*_*N*_(*t*)}, which are the phases describing where each unit is in its firing cycle. The phase synchrony of this system can be measured using the order parameter *r*

$$\begin{array}{c} r=\rho e^{i\psi }=\frac{1}{N}\sum_{n=1}^{N}e^{i\theta_{n}}, \end{array}$$ (1)

where the phase and amplitude of the system are given by *ψ* and *ρ*, respectively. The above definition ensures *ρ* can take values between 0 and 1. Full desynchrony implies *ρ* = 0 while full synchrony is equivalent to *ρ* = 1. We show how synchrony can be related to the envelope amplitude of a signal *f*(*t*) in our previous work [11]. The state of the system can be transformed to a signal representing the neural activity using a superposition of cosine functions

$$\begin{array}{c} f\left(t\right)=\text{Re}\left(r\right)=\frac{1}{N}\sum_{n=1}^{N}\text{cos}\left[\theta_{n}\left(t\right)\right]. \end{array}$$ (2)

The choice of a cosine function is for mathematical convenience since it corresponds to the real part of (1). In addition to this, the cosine function has a maximum at 0, and in classic coupled oscillator models, phase 0 is defined as the phase when neurons produce spikes [25]. Hence post-synaptic potentials in down-stream neurons receiving an input from the modelled population will be a smoothed function of spikes produced in phase 0, so the cosine function captures key features of such post-synaptic potentials. Using the Euler relation and comparing (2) with the real part of (1) shows

$$\begin{array}{c} f(t)=\rho \;\text{cos}(\psi ). \end{array}$$ (3)

We describe the time evolution of the state (for a single population) using the Kuramoto equations, with an additional term describing the effects of stimulation [8, 23]

$$\begin{array}{c} \frac{d\theta_{n}}{dt}=\omega_{n}+\frac{k}{N}\sum_{m=1}^{N}\text{sin}\left(\theta_{m}-\theta_{n}\right)+\hat{V}\left(t\right)Z\left(\theta_{n}\right). \end{array}$$ (4)

The first term of (4) is the natural frequency *ω*_*n*_ which represents the frequency in the absence of external inputs. The second term describes the coupling between the activity of individual units, where *k* is the coupling constant which controls the strength of coupling between each pair of oscillators and hence their tendency to synchronize. The third term describes the effect of stimulation, where the intensity of stimulation is denoted by $\hat{V}(t)$ and the sensitivity to stimulation at a particular phase is denoted by *Z*(*θ*_*n*_). Using the definition of the order parameter given in Eqs (1) and (4) can be transformed to give

$$\begin{array}{c} \frac{d\theta_{n}}{dt}=\omega_{n}+k\rho \;\text{sin}\left(\psi -\theta_{n}\right)+\hat{V}\left(t\right)Z\left(\theta_{n}\right). \end{array}$$ (5)

In this form, it is clear that each oscillator has a tendency to move towards the population phase *ψ* and that the strength of this tendency is controlled by the coupling parameter *k*. An intuition for this behaviour can be obtained using an online simulation of the model [26].

### Response curves

In the previous section we introduced the concept of a neural unit and described the underlying equations governing their dynamics. We now consider the response of these units to stimulation. The unit phase response curve (uPRC), denoted by *Z*(*θ*), describes the sensitivity to stimulation of a neural unit at a particular phase and reflects the observation that the effects of stimulation depend on where a neuron is in its firing cycle [27]. The uPRC is the infinitesimal phase response curve for a neural unit. A strictly positive uPRC, where stimulation can only advance the phase of an oscillator, is referred to as type I. Type II uPRCs have both positive and negative values and hence stimulation can either advance or delay the phase of an oscillator [28]. The uPRC has been found for a variety of systems, including spiking neurons [29], bursting neurons [30] and models of micro-circuits composed of connected populations of excitatory and inhibitory neurons [31]. When such a micro-circuit receives an input, the phase of the oscillations it produces either advances or reduces depending on when within the cycle the input is provided [31]. The effect of weak inputs can be captured using a simple mathematical function *Z*(*θ*). By mapping where a neural unit is in its firing cycle onto a phase variable *θ* ∈ [0, 2*π*], the uPRC describes the change in phase of a single unit due to a stimulus. A general uPRC can be expanded as a Fourier series

$$\begin{array}{c} Z\left(\theta \right)=\frac{a_{0}}{2}+\sum_{m=1}^{\infty }a_{m}\;\text{cos}\left(m\theta \right)+\sum_{m=1}^{\infty }b_{m}\;\text{sin}\left(m\theta \right). \end{array}$$ (6)

The uPRC type is reflected in the zeroth harmonic *a*_0_, or the shift, with |*a*_0_| large and small relative to the other coefficients being indicative of type I and type II curves, respectively. A patient’s response to stimulation can be characterised using the (macroscopic) phase response curve (PRC) and the amplitude response curve (ARC) which respectively describe changes in the phase and amplitude of feedback signals, such as LFP or tremor, at the point of stimulation. Phase oscillator models which incorporate the uPRC can be shown to reproduce the experimentally-known characteristics of this response [11], namely that it should be dependent on both the amplitude and phase of the feedback signals [4, 17, 20].

### Reduced Kuramoto model

In the section ‘The Kuramoto model’ we described the dynamics of a finite system of oscillators using the Kuramoto equations given by Eq (5). In this model, stimulation is described as a perturbation to the phase of an oscillator, with each oscillator experiencing a different effect of stimulation depending on its phase (and determined by *Z*(*θ*)). Stimulation therefore has the effect of changing the distribution of oscillators and hence the order parameter of the system. Since the order parameter, given by Eq (1), is determined by both the amplitude and phase of the system, the expectation is that stimulation will lead to a change in both these quantities, which we refer to as the instantaneous amplitude and phase response of the system. To obtain analytical expressions for these quantities we consider an infinite system of oscillators evolving according to the Kuramoto Eq (5). The distribution of oscillators for this system satisfies the *ansatz* of Ott and Antonsen, which states that the Fourier coefficients of the distribution can be expressed as powers of a single function of frequency and time [32, 33]. Using this, we previously showed [11] that for a general uPRC given by Eq (6) and assuming a Lorentzian distribution for the natural frequencies given by

$$\begin{array}{c} g\left(\omega \right)=\frac{1}{\pi \gamma }[\frac{\gamma^{2}}{{\left(\omega -\omega_{0}\right)}^{2}+\gamma^{2}}], \end{array}$$ (7)

with centre *ω*_0_ and width *γ*, the instantaneous change in the order parameter can be written as

$$\begin{array}{c} \frac{dr}{dt}=\left(i\omega_{0}-\gamma \right)r+\frac{kr}{2}{\left(1-|r\right|}^{2}) \end{array}$$

$$\begin{array}{c} \;+\frac{i\hat{V}\left(t\right)}{2}\{a_{0}r+\sum_{m=1}^{\infty }a_{m}\left[{\left(r^{*}\right)}^{m-1}+r^{m+1}\right]+i\sum_{m=1}^{\infty }b_{m}\left[{\left(r^{*}\right)}^{m-1}-r^{m+1}\right]\}, \end{array}$$ (8)

where $i=\sqrt{-1}$. This leads to expressions for the ARC and PRC due to stimulation

$$\begin{array}{c} \frac{d\rho_{\text{stim}}}{dt}=\frac{\hat{V}\left(t\right)}{2}\left(1-\rho^{2}\right)\sum_{m=1}^{\infty }\rho^{m-1}[a_{m}\;\text{sin}\left(m\psi \right)-b_{m}\;\text{cos}\left(m\psi \right)], \end{array}$$ (9)

and

$$\begin{array}{c} \frac{d\psi_{\text{stim}}}{dt}=\frac{\hat{V}\left(t\right)}{2}\{a_{0}+\left(1+\rho^{-2}\right)\sum_{m=1}^{\infty }\rho^{m}[a_{m}\;\text{cos}\left(m\psi \right)+b_{m}\;\text{sin}\left(m\psi \right)]\}. \end{array}$$ (10)

## Results

### Theory of multi-contact DBS

#### Global and local synchrony

In the section ‘The Kuramoto model’ we defined the order parameter *r* for a single population of phase oscillators with *ρ* as a measure of the phase synchrony. We will now generalise this to the case of *N* oscillators grouped into *S* populations (of variable size) with *N*_*σ*_ oscillators per population. For this system, the order parameter (1) can be rewritten using a double summation

$$\begin{array}{c} r=\frac{1}{N}\sum_{\sigma =1}^{S}\sum_{n=1}^{N_{\sigma }}e^{i\theta_{\sigma n}}, \end{array}$$ (11)

with oscillator *n* of population *σ* being denoted by *θ*_*σn*_. The factor of $\frac{1}{N}$ can be brought inside the first summation and rewritten as $\frac{N_{\sigma }}{N_{\sigma }N}$. Then, with

$$\begin{array}{c} w_{\sigma }=\frac{N_{\sigma }}{N}, \end{array}$$ (12)

the order parameter for the system can be written as

$$\begin{array}{c} r=\sum_{\sigma =1}^{S}\frac{w_{\sigma }}{N_{\sigma }}\sum_{n=1}^{N_{\sigma }}e^{i\theta_{\sigma n}}. \end{array}$$ (13)

Using the definition of the order parameter (1), Eq (13) can be written as

$$\begin{array}{c} r=\sum_{\sigma =1}^{S}w_{\sigma }r_{\sigma }, \end{array}$$ (14)

with

$$\begin{array}{c} \sum_{\sigma =1}^{S}w_{\sigma }=1, \end{array}$$ (15)

and

$$\begin{array}{c} r_{\sigma }=\frac{1}{N_{\sigma }}\sum_{n=1}^{N_{\sigma }}e^{i\theta_{\sigma n}}. \end{array}$$ (16)

Eq (13) shows that the total order parameter *r* for the system can be written as a superposition of the local order parameters *r*_*σ*_ for each population. To clearly distinguish between the two, we will now refer to the total order parameter *r* as the *global* order parameter of the system and *r*_*σ*_ as the *local* order parameter for population *σ*. In each case, the polar representation gives an associated amplitude and phase. The polar representation of the local order parameter can be written in terms of a local amplitude *ρ*_*σ*_ and local phase *ψ*_*σ*_

$$\begin{array}{c} r_{\sigma }=\rho_{\sigma }e^{i\psi_{\sigma }}. \end{array}$$ (17)

Similarly, we refer to *ρ* and *ψ* from (1) as the global amplitude and global phase. The global amplitude (as a measure of total synchrony) is particularly significant since it is correlated to symptom severity in the case of ET and PD. Our objective for stimulation is therefore to maximally reduce the global amplitude *ρ*.

In practice, the global signal may either be measured directly or constructed from LFP recordings. For ET, it is natural to assume that the tremor itself is a manifestation of the global signal. Hence the global signal can be obtained directly by measuring the tremor. The global amplitude and global phase is then taken to be the amplitude and phase of the tremor, respectively. This is of course an idealisation, with the alternative being to correlate pathological neural activity in the LFP with the symptom itself. The global signal would then be constructed using LFP recordings from multiple contacts. We discuss this in more detail in the section entitled ‘Obtaining local activities through electrode measurements’. This approach would be more appropriate in the case of PD, where motor impairment is a set of symptoms whose severity is correlated to LFP activity, particularly in the beta band [4].

We can also relate (14) to feedback signals we might measure by using (2) and taking the real part. Using this, we obtain

$$\begin{array}{c} f\left(t\right)=\sum_{\sigma =1}^{S}w_{\sigma }f_{\sigma }\left(t\right). \end{array}$$ (18)

We refer to *f*(*t*) and {*f*_*σ*_(*t*)} as the global and local signals (or population activities), respectively. Using (3), Eq (18) can also be written in terms of the global and local amplitudes and phases

$$\begin{array}{c} \rho \;\text{cos}\left(\psi \right)=\sum_{\sigma =1}^{S}w_{\sigma }\rho_{\sigma }\;\text{cos}\left(\psi_{\sigma }\right). \end{array}$$ (19)

Using (13), the Kuramoto Eq (4) can also be generalised to multiple populations

$$\begin{array}{c} \frac{d\theta_{\sigma n}}{dt}=\omega_{\sigma n}+\sum_{\sigma^{\prime }=1}^{S}w_{\sigma^{\prime }}k_{\sigma \sigma^{\prime }}\rho_{\sigma^{\prime }}\;\text{sin}\left(\psi_{\sigma^{\prime }}-\theta_{\sigma n}\right)+{\hat{V}}_{\sigma }\left(t\right)Z_{\sigma }\left(\theta_{\sigma n}\right), \end{array}$$ (20)

where ${\hat{V}}_{\sigma }(t)$ is the now the stimulation intensity at a population *σ*. The coupling constant *k* in Eq (4) is now a *S* × *S* matrix with elements *k*_*σσ*′_. The diagonal and off-diagonal elements, denoted by *k*_diag_ and *k*_offdiag_, describe the intrapopulation and interpopulation coupling, respectively.

#### Multi-population response curves

We now derive an expression describing the change in the global amplitude due to stimulation as a function of the local (population) amplitudes and phases. For now it is assumed that the local quantities (to base the stimulation on) can be measured. We will discuss how these quantities can be measured later. Using the polar form of the order parameter (1), Eq (14) can be written as a summation involving the amplitudes and phases of individual populations

$$\begin{array}{c} \rho e^{i\psi }=\sum_{\sigma =1}^{S}w_{\sigma }\rho_{\sigma }e^{i\psi_{\sigma }}. \end{array}$$ (21)

Taking the time derivative of (21) leads to

$$\begin{array}{c} \frac{d\rho }{dt}+i\rho \frac{d\psi }{dt}=\sum_{\sigma =1}^{S}w_{\sigma }[\frac{d\rho_{\sigma }}{dt}+i\rho_{\sigma }\frac{d\psi_{\sigma }}{dt}]e^{i(\psi_{\sigma }-\psi )}, \end{array}$$ (22)

which can be written in terms of the real and imaginary components

$$\begin{array}{c} \begin{array}{cc} \frac{d\rho }{dt} & +i\rho \frac{d\psi }{dt}=\sum_{\sigma =1}^{S}w_{\sigma }\{[\frac{d\rho_{\sigma }}{dt}\;\text{cos}\left(\psi_{\sigma }-\psi \right)-\rho_{\sigma }\frac{d\psi_{\sigma }}{dt}\;\text{sin}\left(\psi_{\sigma }-\psi \right)]\\ & +i[\rho_{\sigma }\frac{d\psi_{\sigma }}{dt}\;\text{cos}\left(\psi_{\sigma }-\psi \right)+\frac{d\rho_{\sigma }}{dt}\;\text{sin}\left(\psi_{\sigma }-\psi \right)]\}. \end{array} \end{array}$$ (23)

It can be seen that the time derivative of the amplitude is the real part of (23)

$$\begin{array}{c} \begin{array}{c} \frac{d\rho }{dt}=\sum_{\sigma =1}^{S}w_{\sigma }[\frac{d\rho_{\sigma }}{dt}\;\text{cos}\left(\psi_{\sigma }-\psi \right)-\rho_{\sigma }\frac{d\psi_{\sigma }}{dt}\;\text{sin}\left(\psi_{\sigma }-\psi \right)]. \end{array} \end{array}$$ (24)

The quantities *dρ*_*σ*_/*dt* and *dψ*_*σ*_/*dt* of Eq (24) are the changes in the amplitude and phase of a population with respect to time. If we assume the distribution of phases within a population satisfies the *ansatz* of Ott and Antonsen [32], we can substitute Eqs (9) and (10) into (24) to obtain the amplitude response due to stimulation in terms of the Fourier coefficients of *Z*(*θ*)

$$\begin{array}{c} \begin{array}{cc} \frac{d\rho_{\text{stim}}}{dt}=\frac{1}{2}\sum_{\sigma =1}^{S}w_{\sigma }{\hat{V}}_{\sigma }\left(t\right)\{ & \sum_{m=1}^{\infty }\rho_{\sigma }^{m-1}[a_{m}^{\left(\sigma \right)}\;\text{sin}\left[\left(m-1\right)\psi_{\sigma }+\psi \right]-b_{m}^{\left(\sigma \right)}\;\text{cos}\left[\left(m-1\right)\psi_{\sigma }+\psi \right]]\\ & -\sum_{m=0}^{\infty }\rho_{\sigma }^{m+1}[a_{m}^{\left(\sigma \right)}\;\text{sin}\left[\left(m+1\right)\psi_{\sigma }-\psi \right]-b_{m}^{\left(\sigma \right)}\;\text{cos}\left[\left(m+1\right)\psi_{\sigma }-\psi \right]]\}. \end{array} \end{array}$$ (25)

Eq (25) contains an expansion over the harmonics of *Z*(*θ*). In our previous paper, we demonstrated that, for a biologically realistic uPRC, it is reasonable to neglect higher harmonic terms (*m* > 1) [11], leading to a simpler expression for the instantaneous amplitude response

$$\begin{array}{c} \begin{array}{cc} \frac{d\rho_{\text{stim}}}{dt}≃\frac{1}{2}\sum_{\sigma =1}^{S}w_{\sigma }{\hat{V}}_{\sigma }\left(t\right)\{[a_{1}^{\left(\sigma \right)}\;\text{sin}\left(\psi \right) & -b_{1}^{\left(\sigma \right)}\;\text{cos}\left(\psi \right)]-\rho_{\sigma }a_{0}^{\left(\sigma \right)}\;\text{sin}\left(\psi_{\sigma }-\psi \right)\\ & -\rho_{\sigma }^{2}\left[a_{1}^{\left(\sigma \right)}\;\text{sin}\left(2\psi_{\sigma }-\psi \right)-b_{1}^{\left(\sigma \right)}\;\text{cos}\left(2\psi_{\sigma }-\psi \right)\right]\}. \end{array} \end{array}$$ (26)

Adaptive coordinated desynchronisation (ACD) is now a strategy that uses Eq (26) to determine when stimulation should be provided. Eq (26) shows the change in the global amplitude due to stimulation can be expressed as a sum of contributions from each population. Each term in the summation can be further split into three terms, the first of which depends only on the global phase with the second and third terms depending on both the global phase and the local quantities. We will refer to these terms as simply the global and local terms, respectively. Eq (26) tells us how the global amplitude (i.e. the symptom severity) should change when stimulation is provided at a particular global phase, local phase and local amplitude. Using this, we can construct a closed-loop strategy for DBS by stimulating only when *dρ*_stim_/*dt* < 0, i.e. when stimulation is predicted to lead to a suppression in the global amplitude.

Eqs (25) and (26) both involve summations over populations, with each term being the product of a weight *w*_*σ*_, a stimulation intensity ${\hat{V}}_{\sigma }$ and a local amplitude response, which we shall denote here by Γ_*σ*_. The local amplitude response Γ_*σ*_ is the contribution of a single population to the global amplitude response. Using Eq (26), Γ_*σ*_ is given by

$$\begin{array}{c} \begin{array}{cc} \Gamma_{\sigma }=[a_{1}^{\left(\sigma \right)}\;\text{sin}\left(\psi \right) & -b_{1}^{\left(\sigma \right)}\;\text{cos}\left(\psi \right)]-\rho_{\sigma }a_{0}^{\left(\sigma \right)}\;\text{sin}\left(\psi_{\sigma }-\psi \right)\\ & -\rho_{\sigma }^{2}\left[a_{1}^{\left(\sigma \right)}\;\text{sin}\left(2\psi_{\sigma }-\psi \right)-b_{1}^{\left(\sigma \right)}\;\text{cos}\left(2\psi_{\sigma }-\psi \right)\right]. \end{array} \end{array}$$ (27)

**Fig 2:**
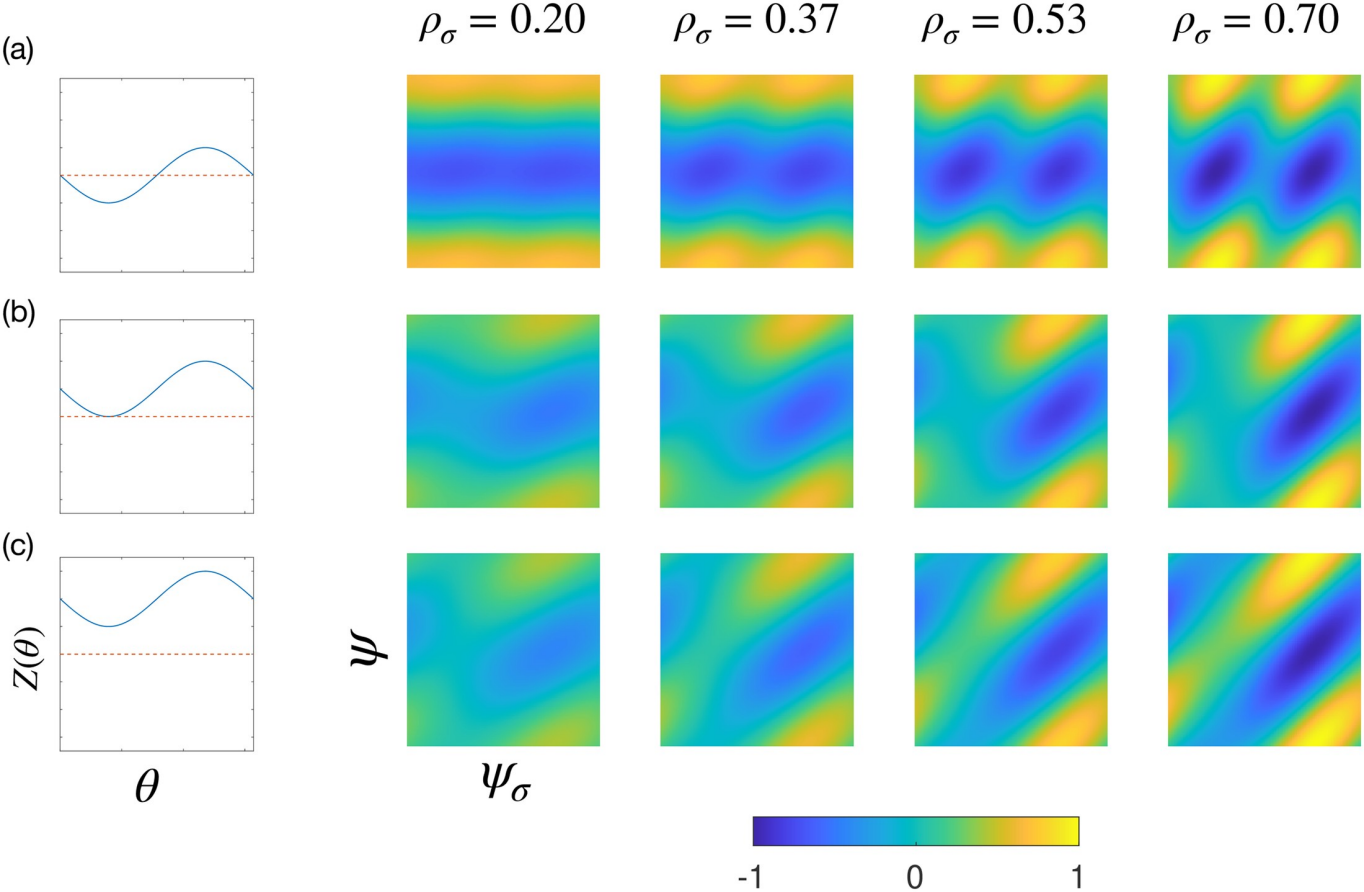
The local amplitude response Γ_*σ*_ for different *ρ*_*σ*_ and *Z*(*θ*). The panels (a), (b) and (c) are for *Z*(*θ*) = *a*_0_/2 − sin(*θ*), with *a*_0_ = 0, 2 and 4, respectively. For each plot, the vertical axis is the global phase (*ψ*) and the horizontal axis is the local (or population) phase (*ψ*_*σ*_). The corresponding uPRC *Z*(*θ*) is also shown, with zero indicated by a red dashed line. Blue regions indicate areas where stimulation is predicted to suppress amplitude.

Plots for Γ_*σ*_ together with the corresponding uPRC for *Z*(*θ*) = *a*_0_/2 − sin(*θ*), with *a*_0_ = 0, 2 and 4 are shown in Fig 2A, 2B and 2C, respectively. Regions in blue are areas of amplitude suppression while orange regions predict amplification. In both cases, these regions can be seen to occur in bands. Graphically, the dependence of Γ_*σ*_ on the global and local phases can be inferred from the direction of the banding. A purely horizontal band implies the response is independent of the local phase. An example of this can be seen at low amplitudes in Fig 2A. Other plots show diagonal banding, which implies the response is dependent on both the global and local phases. This behaviour can be understood by considering the 3 terms of (27). At low amplitudes, the first term dominates, which is only dependent on the global phase. As the local amplitude increases, the second and third terms depending on local quantities become increasingly more important. For the cases where |*a*_0_| is small, the effect is less apparent. The left panel of Fig 2A shows that stimulation can either increase or reduce the phase (i.e. an uPRC of type II), implying a relatively small |*a*_0_|. For this case, the second term can be neglected, leading to a dominance of the first term at low amplitudes where only a small dependence on the local phase can be seen. Fig 2B and 2C shows that stimulation has the effect of only increasing the phase, which is indicative of *Z*(*θ*) with larger |*a*_0_|. For these systems the response can be seen to depend more strongly on the local phase for all amplitudes.

#### Modelling stimulation

The goal of modelling closed-loop DBS due to spatially separated electrodes requires us to consider how electric fields behave within the brain, both due to stimulation and the LFP arising from neural activity. We will consider each of these separately, with the latter being presented in the section entitled ‘Obtaining local activities through electrode measurements’.

Detailed computational models of DBS typically consist of two components: the first is a volume conductor model of the electrodes and the second is a multi-compartmental model of neural populations [34, 35]. When the electrode geometry is considered explicitly, the resulting potential due to stimulation can be found by solving Laplace’s equation using finite element methods [34]. Alternatively, a point source approximation for the electrode may be used, leading to a simple analytical expression for the extracellular potential due to current at the electrode [35]. The effects of stimulation are then calculated using a multi-compartmental neuron, where the dendrites and axons are treated explicitly and then discretised into multiple segments. In this subsection, our aim is to connect these ideas with Eq (25) for the amplitude response. We use the following quantities in this analysis: positions **p**, voltages *V* and currents *I*. Each quantity is accented depending on whether it is associated with an electrode or population, which carry no accent and a hat (ˆ), respectively. A full description of our notation can be found in Table 1.

Firstly, and in contrast to the more detailed models, the response of an individual neural unit is not calculated using the multi-compartmental approach in our model, but instead given a simple analytical form using the uPRC *Z*(*θ*). Then, we expect that for a system of electrodes and neural populations, ${\hat{V}}_{\sigma }$ should depend on the stimulation provided by all the electrodes in the system in addition to the geometry of the electrode placement and properties of the brain tissue. These ideas can be incorporated into our model by simply interpreting the ‘stimulation intensity’ ${\hat{V}}_{\sigma }$ as the extracellular potential (or voltage) at a population due to stimulation. Since Eq (25) describes the response of neural populations, one assumption here is that this potential does not vary within each population, i.e. each unit of population *σ* experiences the same intensity of stimulation. This assumption becomes more valid for ‘small populations’, which can be effectively treated as point sources. We expect the small population assumption to be more valid for systems described by larger *S*. This can be explained by considering a simpler system consisting of 〈*N*_*σ*_〉 units per population and *S* populations. The total number of units is then *N* = 〈*N*_*σ*_〉*S*. For fixed *N*, increasing the number of populations must lead to a reduction in the number of units per population. Since we expect each unit to occupy a volume in space, this therefore leads to smaller populations. Therefore, the small population assumption should be more valid for systems described by larger *S*.

For a system of *L* electrodes and *S* populations, we now express the vector of population voltages $\hat{\text{V}}$ (with elements ${\hat{V}}_{\sigma }$) in terms of currents at the electrode **I**

$$\begin{array}{c} \tilde{D}I=\hat{V}. \end{array}$$ (28)

Both populations and electrodes are now treated as a point sources, with the latter found to be reasonable for modelling stimulation [36]. The currents **I** would be equivalent to the user-controllable stimulation intensities. The elements of matrix $\tilde{\text{D}}$ (of dimensions *S* × *L*) are coefficients ${\tilde{d}}_{\sigma l}$ which reflect the medium and geometry of the electrode-neuron system. For example, if we assume the brain tissue to be a homogeneous isotropic medium (one where the conductivity is independent of both position and direction), the coefficients would be

$$\begin{array}{c} {\tilde{d}}_{\sigma l}=\frac{\kappa_{e}}{|p_{l}-{\hat{p}}_{\sigma }|}, \end{array}$$ (29)

where *κ*_*e*_ is a constant. The positions in space of the electrodes and populations are given by **p**_*l*_ and ${\hat{\text{p}}}_{\sigma }$, respectively. In summary, the quantities we consider in our model for stimulation are voltages ${\hat{V}}_{\sigma }(t)$ at population *σ* due to stimulation which delivers current *I*_*l*_(*t*) to electrode *l*. In the section entitled ‘Obtaining local activities through electrode measurements’ we will also consider a model for measurement, where we use voltages *V*_*l*_(*t*) measured at electrode *l* due to the activity of population *σ* producing currents ${\hat{I}}_{\sigma }(t)$.

#### Optimal stimulation strategy

We now seek an expression for how much current to deliver across each electrode on the basis of feedback signals. A more compact expression for the amplitude response (26) can be written using linear algebra notation, with **Γ** equal to the vector of local amplitude responses (27) and $\hat{\text{V}}$ equal to the vector of voltages at a population

$$\begin{array}{c} \frac{d\rho_{\text{stim}}}{dt}=\frac{1}{2}\left(\Gamma^{⊤}\hat{V}\right), \end{array}$$ (30)

where the weights *w*_*σ*_ have now been absorbed into Fourier coefficients of **Γ** for simplicity. Inserting (28) into (30) leads to an expression for the amplitude response in terms of the currents at the electrodes, i.e. the control variables

$$\begin{array}{c} \frac{d\rho_{\text{stim}}}{dt}=\frac{1}{2}{\left({\tilde{D}}^{⊤}\Gamma \right)}^{⊤}I. \end{array}$$ (31)

Often, concern for tissue damage due to stimulation imposes a limit on how much current can be delivered to a single contact. To account for this, we can also impose a constraint on the current for each contact such that it does not exceed some maximum value *I*_max_

$$\begin{array}{c} 0\leq I\leq I_{\text{max}}. \end{array}$$ (32)

For each time step, our objective is to deliver stimulation which maximally suppresses the global amplitude, i.e. to choose **I**, within the constraints (32), so as to make *dρ*_stim_/*dt* as negative as possible. A simple optimal solution for achieving this can be obtained by setting the current for the *l*th contact to *I*_max_ if the *l*th component of $({\tilde{\text{D}}}^{⊤}\Gamma {)}^{⊤}$ is negative

$$\begin{array}{c} I_{l}=\{\begin{array}{cc} I_{\text{max}} & \text{if}\;{\left({\tilde{D}}^{⊤}\Gamma \right)}_{l}^{⊤}<0\\ 0 & \text{otherwise} \end{array}. \end{array}$$ (33)

It is worth noting that the strategy proposed here is a ‘greedy’ one, where optimality can be locally guaranteed (i.e. per time step), but not overall.

#### The utility of ACD for ET

A number of experimental studies on ET patients have demonstrated the benefit of stimulating according to the phase of the tremor, which we will refer to here as phase-locked (PL) stimulation [17, 19]. Eq (26) predicts this benefit but also predicts this strategy would be suboptimal in general, since the complete amplitude response for a multi-population Kuramoto system requires more knowledge of the patient’s state, involving the local amplitudes and phases. However, under certain conditions, the local terms of Eq (26) may become negligible and hence the predicted amplitude response may approximate to a function of only the global phase. In this scenario, we expect the efficacy difference between ACD and PL stimulation to be negligible. We refer to this efficacy difference here as the utility. To analyse the utility of ACD over PL stimulation we therefore have to understand the significance of the local terms relative to the global terms in (26). It is clear that this significance is dependent both on the parameters of the system and the time dependent state.

Eq (26) contains two local terms involving powers of *ρ*_*σ*_. Since *ρ*_*σ*_ ≤ 1, we expect that the term involving $\rho_{\sigma }^{2}$ will generally be small relative to the other terms. We may then neglect the local term involving $\rho_{\sigma }^{2}$, leading to

$$\begin{array}{c} \begin{array}{cc} \frac{d\rho_{\text{stim}}}{dt}≃\frac{1}{2}\sum_{\sigma =1}^{S}w_{\sigma }{\hat{V}}_{\sigma }\left(t\right)\{[a_{1}^{\left(\sigma \right)}\;\text{sin}\left(\psi \right) & -b_{1}^{\left(\sigma \right)}\;\text{cos}\left(\psi \right)]-\rho_{\sigma }a_{0}^{\left(\sigma \right)}\;\text{sin}\left(\psi_{\sigma }-\psi \right)\}. \end{array} \end{array}$$ (34)

We therefore expect the utility of ACD to be almost entirely dependent on the local term of (34), namely

$$\begin{array}{c} \begin{array}{c} \frac{d\rho_{\text{local}}}{dt}=\frac{1}{2}\sum_{\sigma =1}^{S}w_{\sigma }{\hat{V}}_{\sigma }\left(t\right)\rho_{\sigma }a_{0}^{\left(\sigma \right)}\;\text{sin}\left(\psi_{\sigma }-\psi \right). \end{array} \end{array}$$ (35)

**Fig 3:**
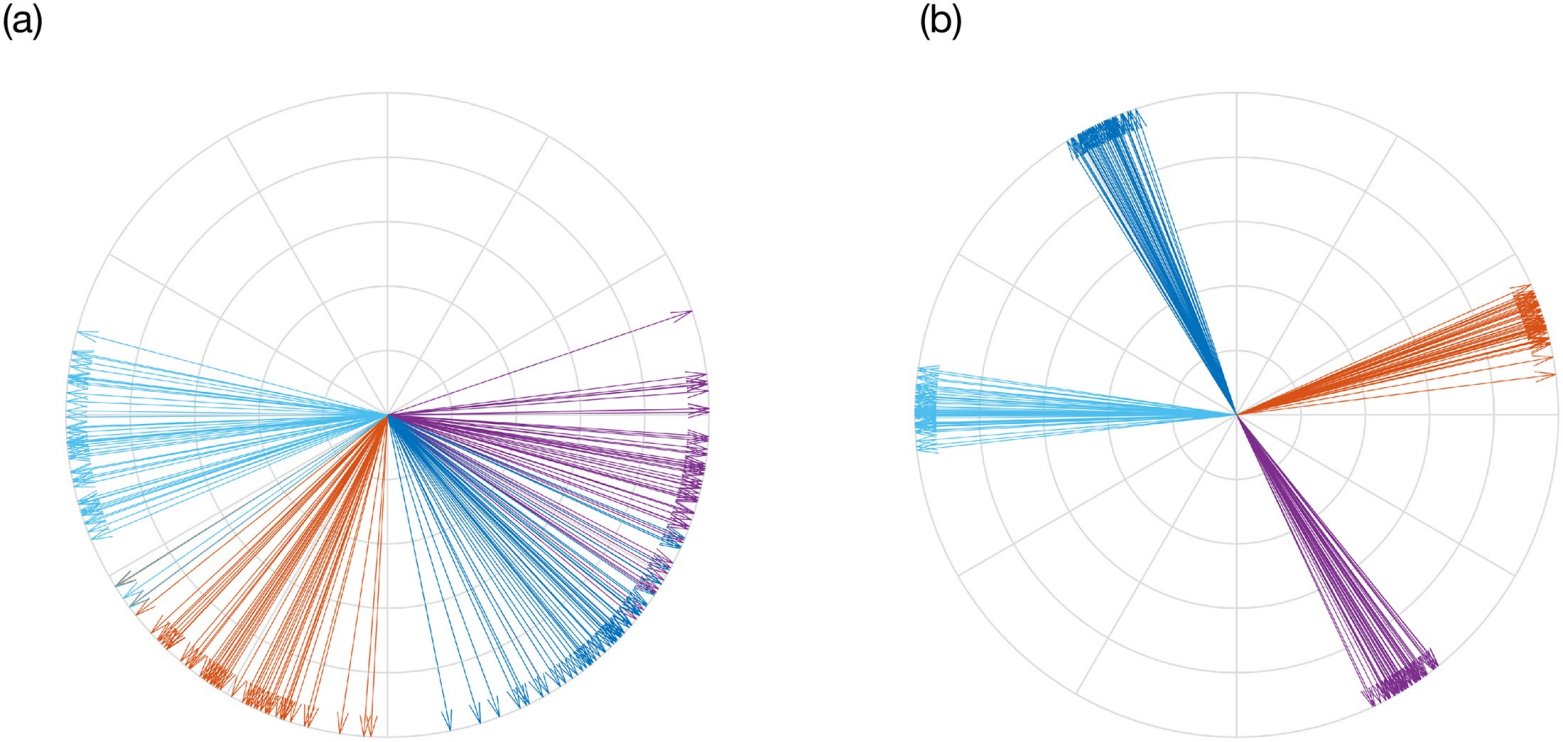
Different configurations of oscillators color coded according to population. Panels (a) and (b) are for a unimodal distribution and multimodal (clustered) distribution, respectively. Configurations were obtained by simulating the multi-population Kuramoto Eq (20).

Hence, we expect the utility of ACD to increase as (35) becomes more significant relative to the other terms in (34). This is likely to occur when the zeroth harmonic of the uPRC $\left|a_{0}^{\left(\sigma \right)}\right|$ is large, implying a type I uPRC. We would expect lower utility for systems where $a_{0}^{\left(\sigma \right)}$ is negligible, i.e. for type II uPRCs. In addition to this, the dependence on sin(*ψ*_*σ*_ − *ψ*) implies that stimulating on the basis of local quantities would only have an effect if the phases of individual populations differ sufficiently from the mean phase. One situation in which such phase difference may be particularly high are for clustered configurations of oscillators. Examples of different configurations of oscillators are shown in Fig 3.

We now investigate which spatial configurations of electrodes and populations should make the effects of stimulation depend only on the global phase. Central to this investigation is an identity which can be obtained by multiplying both sides of Eq (21) by *e*^−*iψ*^ and taking the imaginary part, leading to

$$\begin{array}{c} \text{Im}\;\rho =\sum_{\sigma =1}^{S}w_{\sigma }\rho_{\sigma }\;\text{sin}\left(\psi_{\sigma }-\psi \right)=0. \end{array}$$ (36)

The similarity between this expression and Eq (35) allows us to use (36) to understand the conditions for (35) to be small/negligible. Eq (35) allows for the uPRC type to differ between populations, since the zeroth harmonic is also indexed by *σ*. If we consider the less general case of homogeneous uPRC type, i.e. $a_{0}^{\left(\sigma \right)}=a_{0}$ for all *σ*, then *a*_0_ can be removed from the summation, leading to

$$\begin{array}{c} \begin{array}{c} \frac{d\rho_{\text{local}}}{dt}=\frac{a_{0}}{2}\sum_{\sigma =1}^{S}w_{\sigma }{\hat{V}}_{\sigma }\left(t\right)\rho_{\sigma }\;\text{sin}\left(\psi_{\sigma }-\psi \right). \end{array} \end{array}$$ (37)

For such systems, we can see that if the stimulation intensity at a population is identical across populations, i.e. ${\hat{V}}_{\sigma }(t)=\hat{V}(t)$, then

$$\begin{array}{c} \begin{array}{c} \frac{d\rho_{\text{local}}}{dt}=\frac{\hat{V}\left(t\right)a_{0}}{2}\sum_{\sigma =1}^{S}w_{\sigma }\rho_{\sigma }\;\text{sin}\left(\psi_{\sigma }-\psi \right), \end{array} \end{array}$$ (38)

which equals zero due to (36). Eq (28) relates the current at a particular contact to the stimulation intensity at a population ${\hat{V}}_{\sigma }(t)$ through a matrix which reflects the geometric and electrical properties of the electrode-neuron system. Using (28) for ${\hat{V}}_{\sigma }(t)$ and (37) we can obtain an expression in terms of the current at a contact

$$\begin{array}{c} \begin{array}{c} \frac{d\rho_{\text{local}}}{dt}=\frac{a_{0}}{2}\sum_{l=1}^{L}\sum_{\sigma =1}^{S}{\tilde{d}}_{\sigma l}I_{l}\left(t\right)w_{\sigma }\rho_{\sigma }\;\text{sin}\left(\psi_{\sigma }-\psi \right). \end{array} \end{array}$$ (39)

Eq (39) indicates that, for homogeneous uPRC types, the utility of ACD is dependent on the geometric and electrical properties of the electrode-neuron system. If we consider the case where, for a given electrode, ${\tilde{d}}_{\sigma l}$ does not vary across populations, then this leads to

$$\begin{array}{c} \begin{array}{c} \frac{d\rho_{\text{local}}}{dt}=\frac{a_{0}}{2}[\sum_{l=1}^{L}{\tilde{d}}_{l}I_{l}\left(t\right)]\sum_{\sigma =1}^{S}w_{\sigma }\rho_{\sigma }\;\text{sin}\left(\psi_{\sigma }-\psi \right), \end{array} \end{array}$$ (40)

which also equals zero due to (36). For a system where the matrix elements are given by (29), this condition would be equivalent to one where, for each electrode, the distances between all the populations and the electrode are equal.

In this section we have investigated the utility of ACD with respect to PL stimulation for ET. In summary, we expect the utility to depend on a combination of geometric, uPRC and state related factors. Firstly, we would expect only a small utility for homogeneous isotropic systems where for each electrode the distances between all the populations and the electrode are equal. Secondly, we expect the utility to strongly depend on the uPRC type. For heterogeneous type I systems, we expect the most utility. For type II systems, we expect marginal utility originating from the local term depending on $\rho_{\sigma }^{2}$. Finally, we expect greater utility when ACD is applied to more clustered states of oscillators.

### Numerical simulations

#### Simulated systems

We now test the ACD method and compare its efficacy with CR and PL stimulation. To perform this testing, we simulate the multi population Kuramoto model and use (19) to produce oscillations that are similar to those found in tremor from ET patients. These equations depend on a number of parameters, which together define a particular system in terms of its dynamics, electrode-population configuration and intrinsic response to stimulation *Z*(*θ*). In this subsection, we will describe how systems are generated for our testing.

The dynamics of the system are determined by the parameters of the multi population Kuramoto model, with additional stimulation and noise terms

$$\begin{array}{c} d\theta_{\sigma n}=[\omega_{\sigma n}+\sum_{\sigma^{\prime }=1}^{S}w_{\sigma^{\prime }}k_{\sigma \sigma^{\prime }}\rho_{\sigma^{\prime }}\;\text{sin}\left(\psi_{\sigma^{\prime }}-\theta_{\sigma n}\right)+{\hat{V}}_{\sigma }\left(t\right)Z_{\sigma }\left(\theta_{\sigma n}\right)]dt+\tilde{\sigma }dW, \end{array}$$ (41)

where $\tilde{\sigma }$ is a constant reflecting the amplitude of noise and *W* is a Wiener process. For simplicity, we consider a system of *S* = 3 populations throughout with $Z_{\sigma }(\theta )=a_{0}^{\left(\sigma \right)}/2-\text{sin}(\theta )$. In this form, the uPRC type is determined by a single parameter $a_{0}^{\left(\sigma \right)}$. It is worth emphasising that the results we later present would be the same for any uPRC of the form $Z_{\sigma }(\theta )=a_{0}^{\left(\sigma \right)}/2-\text{sin}(\theta +\phi )$, where *ϕ* is some arbitrary phase shift. The analytical expressions for the response curves are for an infinite system, so we require that *N*_*σ*_ is large. For each population, we choose the number of oscillators *N*_*σ*_ = 600 to satisfy this and to remain computationally feasible.

**Fig 4:**
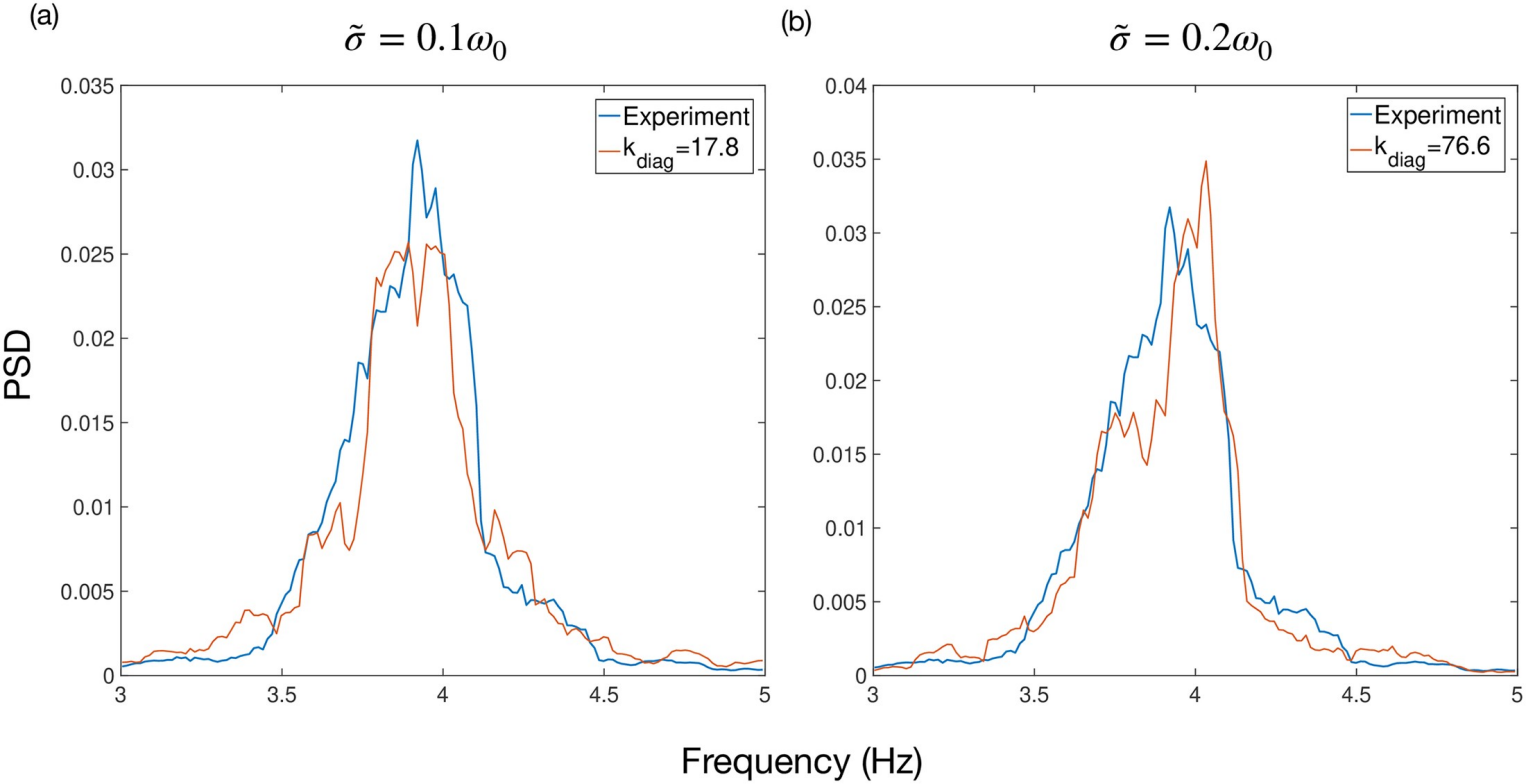
Power spectra of output from the multi population Kuramoto model for different values of $\tilde{\sigma }$. The parameters of the model (41) were chosen to reproduce tremor data from the study of Cagnan et al [17]. In each case, the value of *k*_diag_ was chosen to give the best fit to the power spectrum of a single block from Patient 5’s data.

In order for (41) to produce tremor frequency oscillations, we need to choose an appropriate distribution for the set of natural frequencies {*ω*_*σn*_}. We obtain this by fitting to tremor data from the study of Cagnan et al [17]. In this study, phase-locked DBS was delivered according to tremor in ET patients. This tremor was measured using an accelerometer attached to the patient’s hand. Data was collected from 6 ET patients and 3 dystonic tremor patients. All patients gave their informed consent to take part in the study, which was approved by the local ethics committee in accordance with the Declaration of Helsinki. The data from this study can be obtained through an online repository [37]. The tremor data was filtered using a non-causal Butterworth filter of order 2 with cut-off frequencies at ±2 Hz around the tremor frequency. Stimulation was delivered over a set of trials (typically 9), with each trial consisting of 12 blocks of 5 second phase-locked stimulation at a randomly chosen phase from a set of 12. Each block of phase-locked stimulation was also separated by a 1 second interblock of no stimulation [11]. We assume a Lorentzian form (7) for the distribution of {*ω*_*σn*_} and find the parameters *ω*_0_ and *γ* by fitting to the power spectrum of a single block from Patient 5 of this study. The coefficient of determination for this was found to be *R*^2^ = 0.95, indicating an excellent fit to the data. Using this Lorentzian for the distribution {*ω*_*σn*_}, the multi population Kuramoto model can generate tremor-like output with varying characteristics according to the choice of *k*_diag_ and $\tilde{\sigma }$. Fig 4 shows that, for each value of the noise parameter $\tilde{\sigma }$ we consider in our testing, we can find a value of *k*_diag_ which reproduces the power spectrum of Patient 5. The *R*^2^ for these fits were found to be 0.90 and 0.89 for $\tilde{\sigma }=0.1\omega_{0}$ and $\tilde{\sigma }=0.2\omega_{0}$, respectively. Our goal is to test our methods on a variety of systems so we subsequently test across a range of *k*_diag_.

The response of the system to stimulation (31) also depends on the parameters $\tilde{\text{D}}$, which depend on the geometry of the electrode placement. We assume a homogeneous isotropic system throughout our testing, where the elements of $\tilde{\text{D}}$ are given according to the population and electrode positions

$$\begin{array}{c} {\tilde{d}}_{\sigma l}=\frac{1}{|p_{l}-{\hat{p}}_{\sigma }|}, \end{array}$$ (42)

where we have omitted the constant *κ*_*e*_ for simplicity. Generating relevant configurations also requires an appropriate choice of *L*, which we choose according to a sensible estimate (*L* = 3) of how many contacts can be implanted in the VIM with present generation DBS leads [38–40]. To generate a particular electrode-population configuration, we first approximate the shape of the VIM to be a sphere of unit radius. We then place the coordinates of each electrode to lie on a line across the diameter of this sphere, thus simulating a collinear configuration of contacts commonly found on DBS leads. This VIM-electrode geometry is kept fixed throughout our testing. We place the coordinates of each population according to a ‘configuration parameter’ *η*, which we define in the following way. For each electrode indexed by *l*, we first define *η*_*l*_ as the smallest distance between the electrode and a population divided by the average distance between electrode *l* and a population

$$\begin{array}{c} \eta_{l}=\frac{\min_{\sigma }\left[\right|p_{l}-{\hat{p}}_{\sigma }\left|\right]}{\frac{1}{S}\sum_{\sigma =1}^{S}\left|p_{l}-{\hat{p}}_{\sigma }\right|}. \end{array}$$ (43)

The parameter *η*_*l*_ takes values between 0 and 1 and describes how localised the effects of stimulation are from a single electrode. To obtain the configuration parameter for the system, we average over electrodes

$$\begin{array}{c} \eta =\frac{1}{L}\sum_{l=1}^{L}\eta_{l}. \end{array}$$ (44)

**Fig 5:**
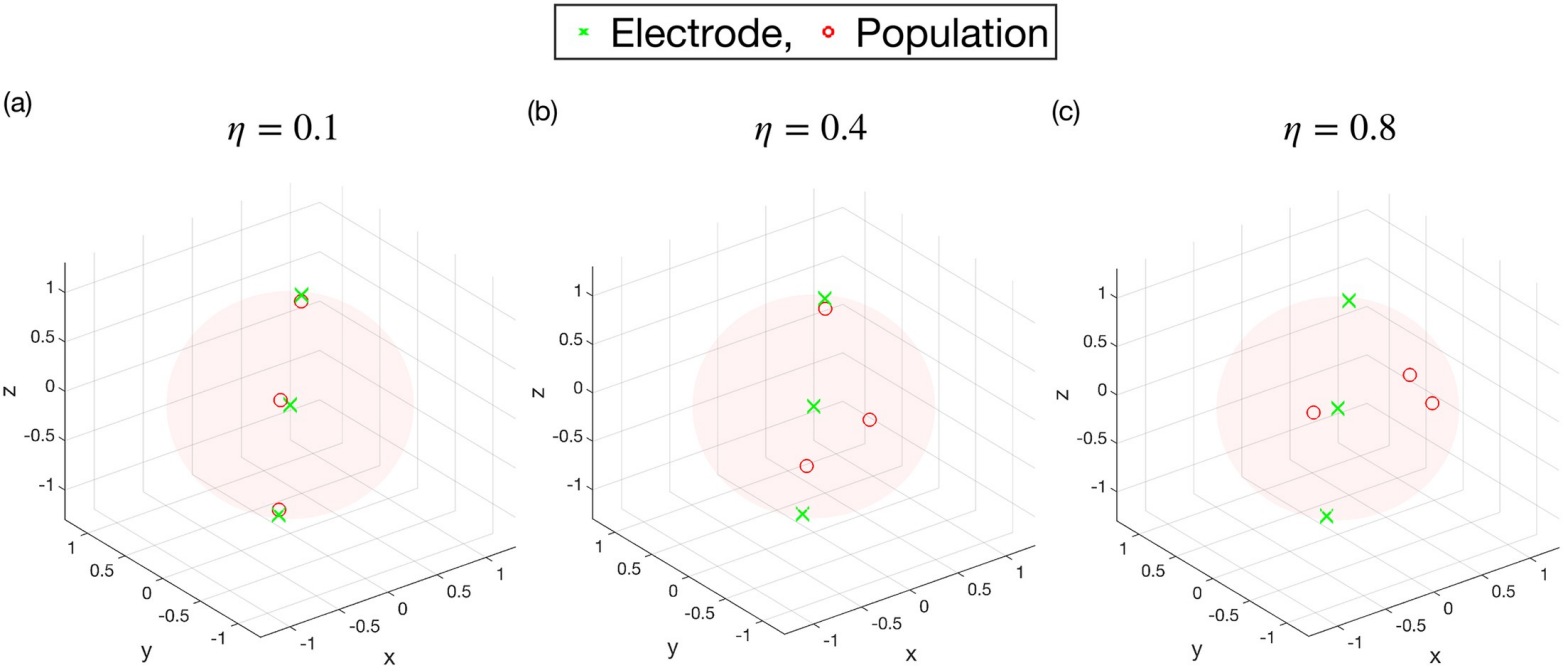
Visualisations of 3 electrode 3 population systems for different values of the configuration parameter *η*. The 3 electrodes lie on a straight line across a sphere of unit diameter. Panel (a) shows a configuration for *η* = 0.1, where each population is placed very close to an electrode. Panels (b) and (c) show systems for *η* = 0.4 and *η* = 0.8, respectively, where the populations are more dispersed relative to the electrodes. In this scenario, stimulation from electrodes may affect multiple populations.

A small *η* means the effects of stimulation are localised to a particular population, as shown in Fig 5A. When *η* approaches one, the smallest distance between an electrode and a population becomes equal to the average, meaning the effects of stimulation become delocalised. This is shown in Fig 5B and 5C.

Due to (28), the stimulation intensity ${\hat{V}}_{\sigma }$ is dependent on both the geometry of the system and the electrode current *I*_*l*_. This can lead to large values for ${\hat{V}}_{\sigma }$ if, for example, the separation between populations and electrodes becomes small. To avoid this, we can define the stimulation intensity in terms of the maximum perturbation to a single oscillator Δ*θ*_max_. Using Eq (41), the change in *θ*_*σn*_ due to stimulation over a time step in simulation Δ*t* can be expressed as

$$\begin{array}{c} \Delta \theta_{\sigma n}={\hat{V}}_{\sigma }\left(t\right)Z_{\sigma }\left(\theta_{\sigma n}\right)\Delta t. \end{array}$$ (45)

Inserting (28) into (45) leads to

$$\begin{array}{c} \Delta \theta_{\sigma n}=[\sum_{l=1}^{L}{\tilde{d}}_{\sigma l}I_{l}\left(t\right)]Z_{\sigma }\left(\theta_{\sigma n}\right)\Delta t. \end{array}$$ (46)

If we assume a system where the uPRC are the same across populations and normalised to 1, then Δ*θ*_max_ can be expressed in terms of the maximum current at an electrode

$$\begin{array}{c} I_{\text{max}}=\frac{\Delta \theta_{\text{max}}}{\max_{\sigma }[\sum_{l=1}^{L}{\tilde{d}}_{\sigma l}]\Delta t}. \end{array}$$ (47)

The *S* × *S* coupling constant matrix can be simplified by focussing only on the diagonal and off-diagonal components, which we denote by *k*_diag_ and *k*_offdiag_, respectively. We fix *k*_offdiag_ = 0, so that *k*_diag_ can be used to control the level of clustering for a particular configuration of oscillators, with increasing *k*_diag_ leading to increasingly multi-modal distributions of oscillators.

#### Running the simulation

Our aim is to compare the efficacies of CR, PL and ACD for a variety of test systems. We define a system according to a set of parameters $\left\{\tilde{\sigma },k_{\text{diag}},\eta ,a_{0},\Delta \theta_{\text{max}}\right\}$, which can be used to change both the dynamics of the system and its response to stimulation. The high dimensionality of this space prevents a full exploration so we instead investigate the efficacy of each method as a function of a single parameter, leaving the remaining parameters fixed. In our testing we define the efficacy of a particular DBS strategy to be its desynchronising effect on a system of coupled oscillators. We measure this by averaging *ρ* for a period of time after stimulation is applied and then averaging this over *n*_trials_ trials to obtain $\bar{\rho }$. For each trial, we randomly generate a system with a given value of *η* and then integrate (41) using Euler’s method with a time step of Δ*t* = 0.0025 for a total of 15 seconds. After 5 seconds, we turn on the stimulation and then after 10 seconds we begin averaging *ρ* for the remaining 5 seconds. A summary of the parameters common to all our simulations, unless stated otherwise, are provided in Table 2. The error bars in subsequent plots are the standard errors when averaged over *n*_trials_ trials.

**Table 2:** Summary of parameters common to all simulations, unless stated otherwise.

Parameter	Value	Description
Δ*t*	0.0025	Integration time step
*T*	15	Simulation time
*t*_*start*_	5	Stimulation start time
*t*_*avg*_	10	Start time for averaging *ρ*
*L*	3	Number of electrodes
*S*	3	Number of populations
*N*_*σ*_	600	Number of oscillators per population
*k*_offdiag_	0	Off-diagonal of coupling constant matrix
*a*_1_	0	First even Fourier coefficient
*b*_1_	-1	First odd Fourier coefficient
*n*_trials_	80	Number of trials
*f*_max_	130	Maximum frequency for PL stimulation
*f*_burst_	3.92	CR burst frequency
*f*_train_	130	CR HF train frequency
*t*_burst_	0.1	CR burst time
*ω*_0_/2*π*	3.92	Centre of natural frequency distribution
*γ*/2*π*	0.15	Width of natural frequency distribution

We use the time-shifted variant of CR neuromodulation [8, 41] in our testing where stimulation is delivered in bursts of HF pulse trains. The stimulation pattern is time-shifted across each electrode indexed by *l* by

$$\begin{array}{c} \tau_{l}=\frac{2\pi }{\bar{\omega }L}\left(l-1\right), \end{array}$$ (48)

where $\bar{\omega }$ is the mean of the natural frequencies, which we take to be *ω*_0_. The number of bursts per second, the burst frequency *f*_burst_, was chosen to be equal to $\bar{\omega }/2\pi$ and the HF pulse train frequency *f*_train_ was chosen to be 130 Hz. The width of each burst *t*_burst_ was chosen to be 0.1 seconds. We implement PL stimulation by neglecting the local terms of Eq (26), leading to

$$\begin{array}{c} \begin{array}{cc} \frac{d\rho_{\text{PL}}}{dt}=\frac{1}{2}\sum_{\sigma =1}^{S}w_{\sigma }{\hat{V}}_{\sigma }\left(t\right)\{[a_{1}^{\left(\sigma \right)}\;\text{sin}\left(\psi \right) & -b_{1}^{\left(\sigma \right)}\;\text{cos}\left(\psi \right)]\}. \end{array} \end{array}$$ (49)

Stimulation is then provided when $\frac{d\rho_{\text{PL}}}{dt}<0$, with the resulting strategy depending only on the global phase *ψ*.

**Fig 6:**
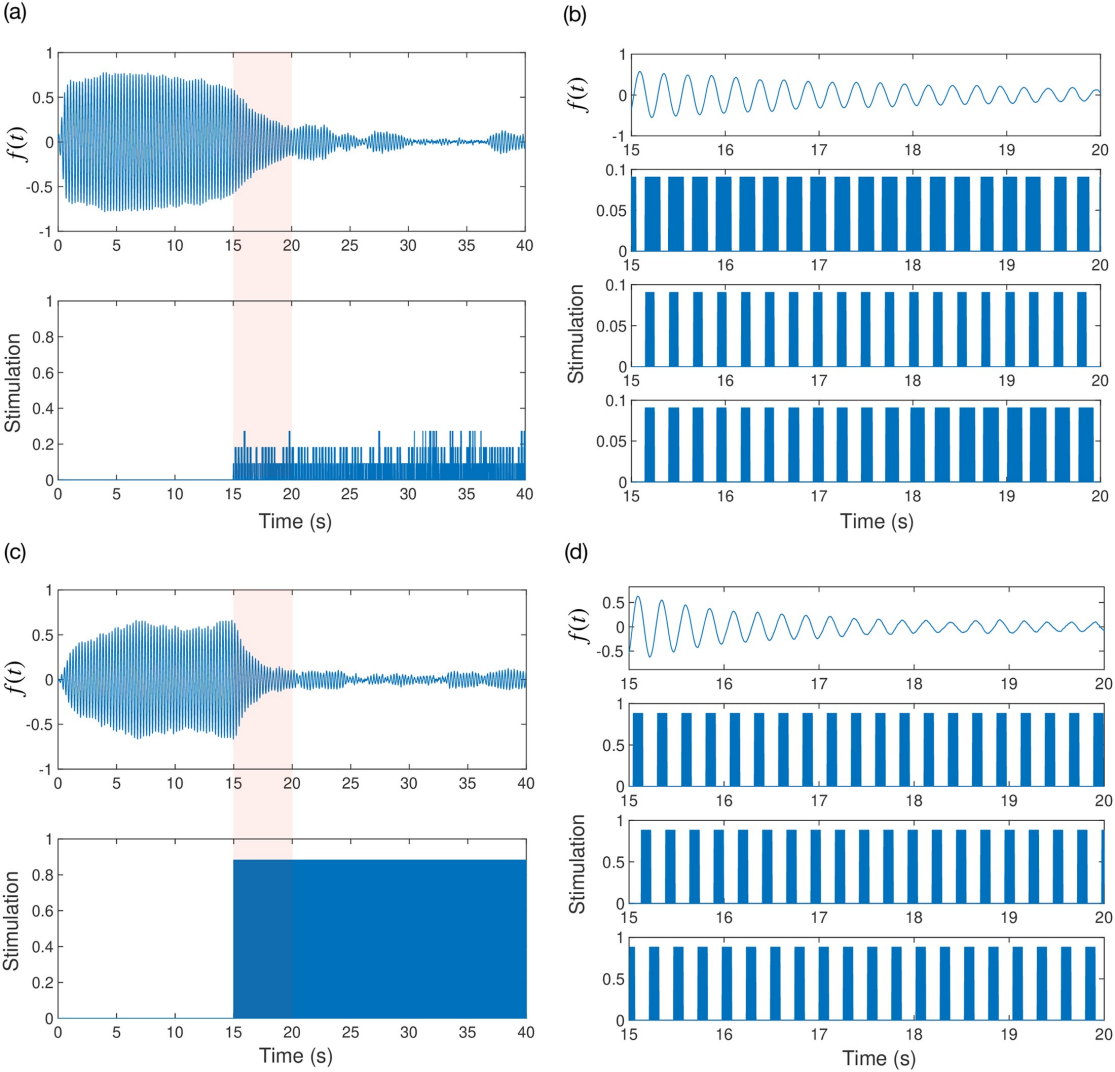
Output from numerical simulations showing the effects of adaptive coordinated desynchronisation (ACD) and coordinated reset (CR). The top panels (a)-(b) show the model output for ACD and the bottom panels (c)-(d) show the model output for CR. The left column shows model output together with the stimulation (averaged across contacts) as a function of time. The right column shows model output and stimulation for each contact for the respective shaded segment of the left column. The shaded portion of the stimulation for the left column is simply the average across contacts of the stimulation shown in the right column.

Example output showing the desynchronising effects of ACD and CR are shown in Fig 6 for a system with parameters ($\eta =0.1,k_{\text{diag}}=55,\tilde{\sigma }=0.1\omega_{0},a_{0}=1)$. Here, the system is simulated for a longer period of *T* = 40 seconds and stimulation is turned on at *T* = 15 seconds. Fig 6A and 6B shows output from ACD while Fig 6C and 6D shows output from CR. To demonstrate the effect of CR we used a higher stimulation intensity of Δ*θ*_max_ = 0.01*π* compared to Δ*θ*_max_ = 0.001*π* for ACD. Comparing the outputs from ACD and CR, it is clear that the stimulation pattern from ACD is significantly different from that produced by CR, with the latter pattern being simply time-shifted across electrodes. The stimulation pattern from ACD allows for the possibility that multiple electrodes may be stimulated simultaneously. Fig 6 also shows that ACD achieves a similar desynchronising effect to CR whilst using a much lower stimulation intensity.

#### Efficacy and efficiency vs stimulation intensity

**Fig 7:**
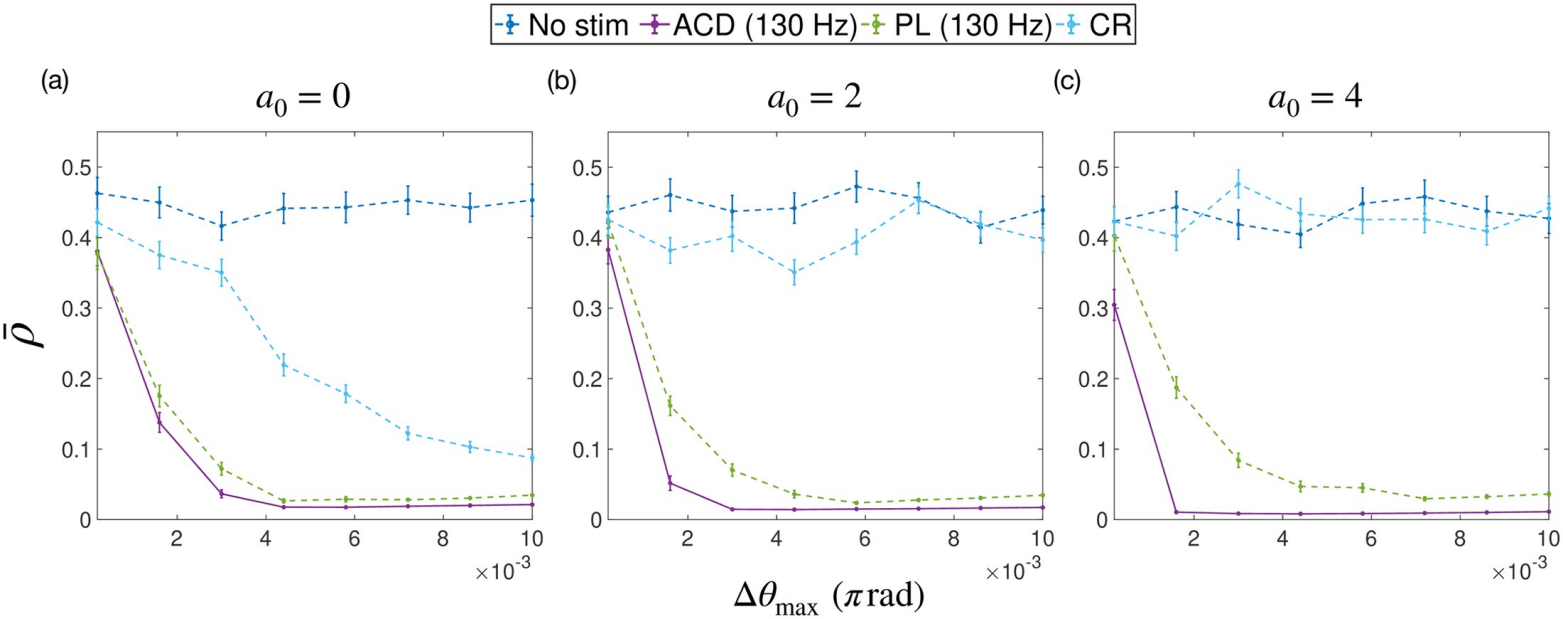
The average amplitude of simulated data $\bar{\rho }$ as a function of the stimulation intensity Δ*θ*_max_. The strategies tested were: no stimulation (no stim), adaptive coordinated desynchronisation (ACD), phase-locked (PL) and coordinated reset (CR). The average amplitude is a measurement of efficacy, where lower amplitudes indicate higher efficacy. The maximum stimulation frequencies used for ACD and PL are also given in the legend. Each sub plot shows a set of simulations performed with a particular zeroth harmonic of the uPRC *a*_0_.

**Fig 8:**
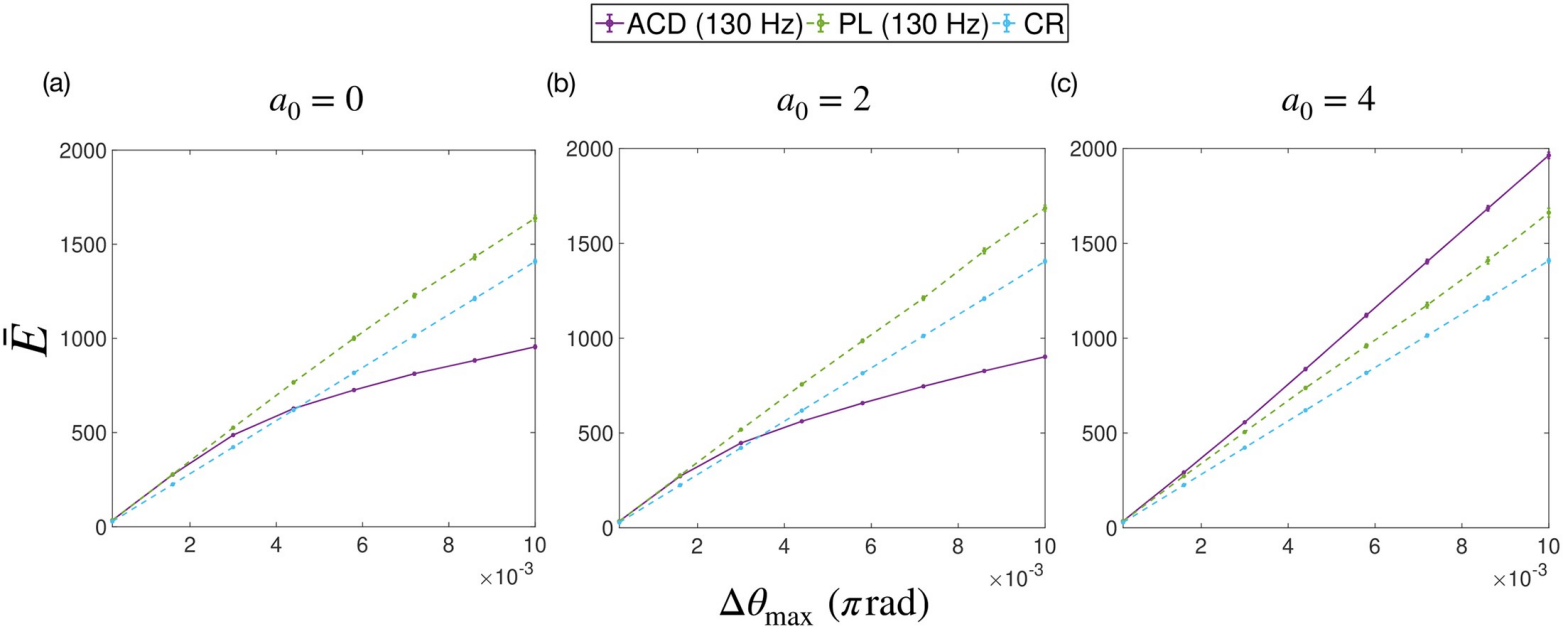
The total energy used by different stimulation strategies, when applied to a simulated system, as a function of the stimulation intensity Δ*θ*_max_. The strategies tested were: adaptive coordinated desynchronisation (ACD), phase-locked (PL) and coordinated reset (CR). The maximum stimulation frequencies used for ACD and PL are also given in the legend. Each sub plot shows a set of simulations performed with a particular zeroth harmonic of the uPRC *a*_0_.

Figs 7 and 8 show the efficacy and efficiency of each stimulation strategy as a function of the stimulation intensity Δ*θ*_max_, respectively. The uPRC was assumed to be homogeneous across populations, i.e. *Z*_*σ*_(*θ*) = *a*_0_/2 − sin(*θ*). Each sub plot shows a set of simulations performed with a particular zeroth harmonic of the uPRC *a*_0_. We fixed *η* = 0.1, $\tilde{\sigma }=0.1\omega_{0}$ and *k*_diag_ = 55 in all cases and then performed our investigations using 3 values of *a*_0_ (*a*_0_ = 0, *a*_0_ = 2 and *a*_0_ = 4).

Fig 7A shows results for *a*_0_ = 0, corresponding to a type II uPRC. For PL and ACD, a regime exists where the efficacy of each strategy is seen to increase with increasing Δ*θ*_max_. The utility of ACD is found to be marginally higher than PL, with the exception being at low intensities (approximately Δ*θ*_max_ ≤ 0.0005*π*), where both efficacies are approximately equal. The efficacy of CR is shown to increase across the range of Δ*θ*_max_ tested, leading to a reduced utility of ACD over this method with increasing Δ*θ*_max_. Increasing *a*_0_ leads to a reduced efficacy for CR, which is clearly shown in Fig 7B and 7C. The increased utility of ACD with increasing *a*_0_, as predicted in the section ‘The utility of ACD for ET’, can also be seen, although this utility is also seen to diminish with increasing Δ*θ*_max_.

The total amount of stimulation delivered by each strategy (the sum of all stimulation pulses) can be likened to the ‘total energy’ used by a strategy. We denote this quantity by $\bar{E}$. It is expected that for open loop methods, such as CR, $\bar{E}$ should increase linearly with Δ*θ*_max_. However, for closed-loop methods, where the stimulation pattern is dependent on the signal itself, a more complex relationship is likely to exist. Plots for $\bar{E}$ as a function of Δ*θ*_max_ are shown in Fig 8. A linear relationship for $\bar{E}(\Delta \theta_{\text{max}})$ is found for the case of CR (as expected). In this case of PL stimulation, $\bar{E}(\Delta \theta_{\text{max}})$ is shown to be approximately linear across Δ*θ*_max_. For ACD, a linear regime is observed for small Δ*θ*_max_, where $\bar{E}(\Delta \theta_{\text{max}})$ for ACD and PL stimulation are approximately equal. For *a*_0_ = 0 and *a*_0_ = 2, $\bar{E}(\Delta \theta_{\text{max}})$ then begins to plateau. In these cases, $\bar{E}(\Delta \theta_{\text{max}})$ for ACD is predicted to be lower than both PL and CR for larger Δ*θ*_max_. We find a qualitatively different relationship for *a*_0_ = 4, where $\bar{E}(\Delta \theta_{\text{max}})$ for ACD is found to be approximately linear across the range. For this case, $\bar{E}(\Delta \theta_{\text{max}})$ for ACD is predicted to be higher than both PL and CR at larger Δ*θ*_max_.

In subsequent testing we ensure that, for a given trial, the amount of stimulation delivered by a particular strategy is approximately equal. To achieve this, we restrict our testing to small Δ*θ*_max_, where $\bar{E}(\Delta \theta_{\text{max}})$ is approximately equal for both ACD and PL. Smaller Δ*θ*_max_ is also more relevant from a practical standpoint, since lower stimulation intensities are better for power consumption and reducing the side effects of DBS. We also apply a correction to Δ*θ*_max_ for CR, using the relationships found in Fig 8. This amounts to using a Δ*θ*_max_ for CR of ≃ 1.3 times that of ACD.

#### Efficacy vs coupling

**Fig 9:**
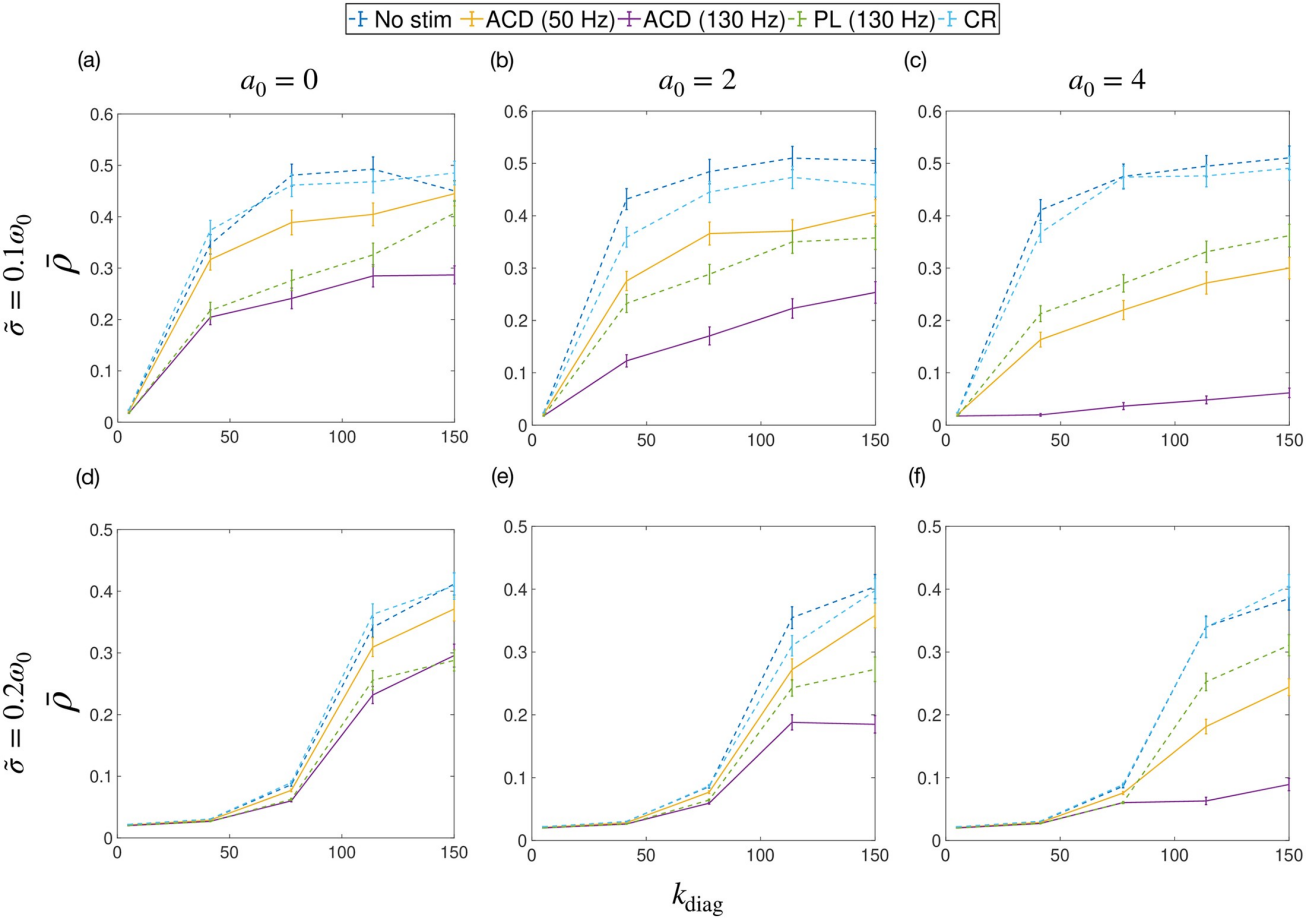
The average amplitude of simulated data $\bar{\rho }$ as a function of the coupling constant *k*_diag_. The strategies tested were: no stimulation (no stim), adaptive coordinated desynchronisation (ACD), phase-locked (PL) and coordinated reset (CR). The average amplitude is a measurement of efficacy, where lower amplitudes indicate higher efficacy. The maximum stimulation frequencies used for ACD and PL are also given in the legend. Solid lines are for the ACD method. Each sub plot shows a set of simulations performed with a particular zeroth harmonic of the uPRC *a*_0_ and value of noise $\tilde{\sigma }$.

Fig 9 shows the efficacy of various stimulation strategies as a function of *k*_diag_. The uPRC was assumed to be homogeneous across populations, i.e. *Z*_*σ*_(*θ*) = *a*_0_/2 − sin(*θ*). Each sub plot shows a set of simulations performed with a particular zeroth harmonic of the uPRC *a*_0_ and value of noise $\tilde{\sigma }$. We fixed *η* = 0.1 and Δ*θ*_max_ = 0.001*π* in all cases. We performed our investigations using 2 values of $\tilde{\sigma }$ ($\tilde{\sigma }=0.1\omega_{0}$ and $\tilde{\sigma }=0.2\omega_{0}$) and 3 values of *a*_0_ (*a*_0_ = 0, *a*_0_ = 2 and *a*_0_ = 4). ACD was tested at maximum frequencies of 130 Hz and 50 Hz.

Significant improvements with ACD at 130 Hz over PL and CR can be observed when *a*_0_ = 2 and *a*_0_ = 4 (i.e. type I uPRCs) for both noise parameters tested. The utility of ACD over other methods is also shown to be greatest when *k*_diag_ is larger, which corresponds to larger local amplitudes *ρ*_*σ*_ and increased clustering. The increased utility of ACD with increasing *a*_0_ and clustering agree with our predictions in the section ‘The utility of ACD for ET’. For systems where *a*_0_ = 0, ACD is found to be marginally more effective than PL, as predicted. ACD at 50 Hz is generally found to have similar efficacy to PL at 130 Hz but with stimulation delivered at half the frequency, translating to considerably less energy consumption.

#### Efficacy vs configuration parameter

We now investigate the efficacy of each stimulation strategy for different values of the configuration parameter *η*. In the section ‘The utility of ACD for ET’ we predicted that the efficacy of ACD should depend explicitly on the geometry of the electrode-population configuration and that the utility of ACD relative to PL stimulation should diminish as *η* increases. We also predicted that this effect could be mitigated if the uPRCs for the system of populations were heterogeneous in type, i.e. by using a form $Z_{\sigma }(\theta )=a_{0}^{\left(\sigma \right)}/2-\text{sin}(\theta )$.

**Fig 10:**
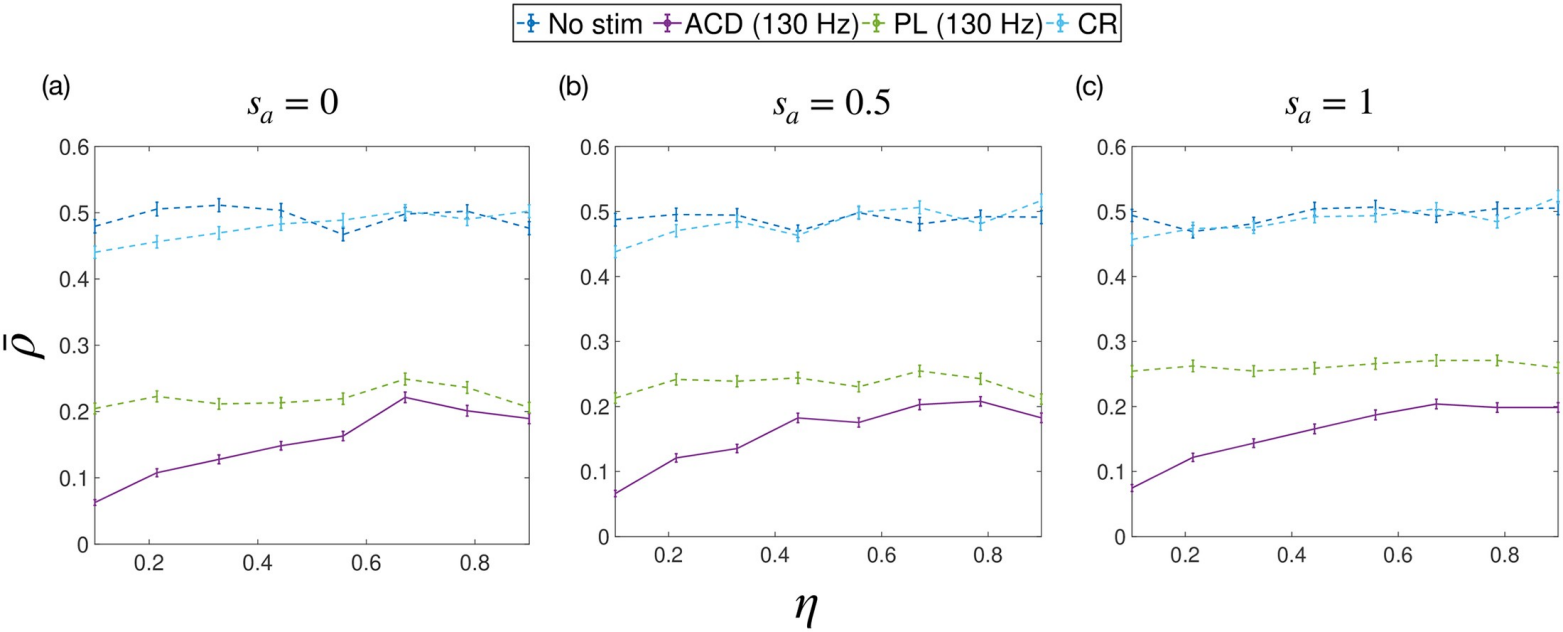
The average amplitude of simulated data $\bar{\rho }$ as a function of the configuration parameter *η*. The strategies tested were: no stimulation (no stim), adaptive coordinated desynchronisation (ACD), phase-locked (PL) and coordinated reset (CR). The average amplitude is a measurement of efficacy, where lower amplitudes indicate higher efficacy. The maximum stimulation frequencies used for ACD and PL are also given in the legend. Each sub plot shows a set of simulations performed with a particular value for the standard deviation of the zeroth harmonic of the uPRC *s*_*a*_.

To test these predictions, we investigated the efficacy of each stimulation strategy as a function of *η* by simulating (41) with *k*_diag_ = 55, $\tilde{\sigma }=0$ and Δ*θ*_max_ = 0.0014*π*. To investigate the effects of heterogeneous $a_{0}^{\left(\sigma \right)}$ we generate each system of 3 populations with $a_{0}^{\left(\sigma \right)}$ sampled from a normal distribution with mean 2 and standard deviation *s*_*a*_. We performed our investigations at 3 values of *s*_*a*_ (*s*_*a*_ = 0, *s*_*a*_ = 0.5 and *s*_*a*_ = 1), corresponding to the homogeneous case and then increasing heterogeneity, respectively. For each *η*, we randomly generate a set of 480 systems, calculate the efficacy of each strategy and then average across all systems. The results from these simulations are shown in Fig 10, with each sub plot showing a set of simulations performed with a particular *s*_*a*_. Fig 10 shows that the utility of ACD relative to PL stimulation reduces with increasing *η*, but that this effect is reduced as the level of heterogeneity in the uPRC type increases. This is in agreement with our predictions from the section ‘The utility of ACD for ET’.

### Obtaining local activities through electrode measurements

We have shown that in order to optimally desynchronise a system of Kuramoto oscillators, stimulation should be provided on the basis of the state, which includes the global phase *ψ* and local quantities {*ψ*_*σ*_} and {*ρ*_*σ*_}, reflecting the population level activity. In the case of ET, we expect the global phase to be measurable through the tremor but we have not yet described how the local quantities should be determined. In this section, we will describe how this might be achieved using LFP measurements through different contacts. Our goal here is not to construct a detailed electrophysiological model of neural activity but instead to outline the various assumptions required to resolve the local state. As before, we will use the following quantities in this analysis: positions **p**, voltages *V* and currents *I*. We now distinguish quantities associated with neural unit compartments using a dash (´).

The extracellular LFP due to neural activity arises from transmembrane currents in the vicinity of the electrode [42]. Modelling the LFP can be achieved using a multi-compartmental representation of each neuron, where the axons and dendrites are treated explicitly and discretised into multiple segments (or compartments). Each compartment is then effectively treated as a point current source in space [34, 35, 42]. Using this, we now consider a multi-population system where the *n*th neural unit of population *σ* is discretised into *M*_*σn*_ compartments, with each compartment indexed by *m* and at a position denoted by ${\overset{´}{\text{p}}}_{\sigma nm}$. A general expression for the voltage at a point electrode can then be written in terms of the current at a compartment *Í*_*σnm*_(*t*)

$$\begin{array}{c} V_{l}\left(t\right)=\sum_{\sigma =1}^{S}\sum_{n=1}^{N_{\sigma }}\sum_{m=1}^{M_{\sigma n}}d\left(p_{l},{\overset{´}{p}}_{\sigma nm}\right){\overset{´}{I}}_{\sigma nm}\left(t\right), \end{array}$$ (50)

where $d({\text{p}}_{l},{\overset{´}{\text{p}}}_{\sigma nm})$ are coefficients which reflect the medium and geometry of the system. We now let ${\overset{´}{\text{p}}}_{\sigma nm}={\hat{\text{p}}}_{\sigma }+\Delta {\overset{´}{\text{p}}}_{\sigma nm}$, i.e. we now define a vector to a compartment in terms of a vector to a region (or population) plus a shift. As before, we assume ‘small populations’, i.e. the region $\Delta {\overset{´}{\text{p}}}_{\sigma nm}$ is small relative to ${\hat{\text{p}}}_{\sigma }$, then we can write

$$\begin{array}{c} V_{l}\left(t\right)=\sum_{\sigma =1}^{S}d\left(p_{l},{\hat{p}}_{\sigma }\right){\hat{I}}_{\sigma }\left(t\right), \end{array}$$ (51)

where the double summation over neurons and compartments is the population activity

$$\begin{array}{c} {\hat{I}}_{\sigma }\left(t\right)=\sum_{n=1}^{N_{\sigma }}\sum_{m=1}^{M_{\sigma n}}{\overset{´}{I}}_{\sigma nm}\left(t\right). \end{array}$$ (52)

Since we are dealing with oscillations, we can give a general form to ${\hat{I}}_{\sigma }(t)$ in terms of the local phases and amplitudes

$$\begin{array}{c} {\hat{I}}_{\sigma }\left(t\right)=c\rho_{\sigma }\;\text{cos}\left(\psi_{\sigma }\right), \end{array}$$ (53)

where *c* is a constant. The potential at the electrodes (51) can then be written in matrix form

$$\begin{array}{c} (\begin{array}{ccccc} d_{11} & d_{12} & d_{13} & \ldots & d_{1S}\\ \vdots & \ddots & & & \\ \vdots & & \ddots & & \\ \vdots & & & \ddots & \\ d_{L1} & & & & d_{LS} \end{array})(\begin{array}{c} \rho_{1}\;\text{cos}\left(\psi_{1}\right)\\ \rho_{2}\;\text{cos}\left(\psi_{2}\right)\\ \rho_{3}\;\text{cos}\left(\psi_{3}\right)\\ \vdots \\ \rho_{S}\;\text{cos}\left(\psi_{S}\right) \end{array})=(\begin{array}{c} V_{1}\left(t\right)\\ V_{2}\left(t\right)\\ V_{3}\left(t\right)\\ \vdots \\ V_{L}\left(t\right) \end{array}), \end{array}$$ (54)

where for simplicity we have denoted $d_{l\sigma }=cd({\text{p}}_{l},{\hat{\text{p}}}_{\sigma })$. Eq (54) can be expressed in a more compact form with **D** denoting the matrix of coefficients (of dimensions *L* × *S*), **f** as the vector of neural activities and **V** as the vector of electrode measurements.

$$\begin{array}{c} Df=V. \end{array}$$ (55)

Eq (55) relates the voltages at the electrodes **V** to the neural activities **f**. Eq (54) shows that what we actually measure at the electrodes is a linear superposition of population activities. We may also include a ‘stimulation artefact’, which arises due to the electrodes recording the stimulation pulses themselves. We do not consider this here for simplicity, but discuss it briefly in the section ‘Limitations and future work’. For the cases where **D** is approximately diagonal, the population quantities could be accurately recovered (although *ρ*_*σ*_ would be scaled). Such cases would represent systems consisting of small separated regions of activity, with each electrode positioned close to each region (see Fig 5A).

Methods such as independent component analysis (ICA) [43] are well-suited to solving the general problem of recovering a vector of ‘source signals’ **f**(*t*) (in this case the population activities) given a vector of recordings **V**(*t*), as expressed in Eq (54), although the method cannot recover the scaling. In theory the matrix **D**, which depends on the medium and geometry of the system, should not evolve with time. We therefore envisage ICA being applied offline to recover **D** and then used to obtain the local signals. The goal of ICA here is to resolve the *S* population quantities from *L* electrode measurements. The determined case (*S* = *L*) is perhaps the most common and more easily solved since the mixing matrix **D** can be inverted. If we assume the case of *S* = *L*, then ICA will always resolve exactly *L* components. With this assumption, increasing the number of electrodes in a system has a definite purpose: it increases our potential to resolve the internal state. Assuming a larger number of populations also increases the validity of the small region approximation and thus the accuracy of ACD. It may also be possible to obtain good approximations to the state by using *L* < *S* electrodes, since in some cases the weights *w*_*σ*_ may be small for some populations and can hence be neglected. Once the vector of local signals have been resolved using ICA, the global signal can then be constructed using Eq (18). This would involve choosing a set of weights {*w*_*σ*_} such that the resulting global amplitude is correlated to the symptom severity.

## Discussion

We have presented a new method of closed-loop DBS designed for systems which use multiple independently powered contacts. Unique to our work is the formulation of a stimulation strategy for multiple spatially separated populations of coupled oscillators. We use these systems to model synchronous activity, which manifests in LFP recordings and is linked to the severity of a number of neurological disorders. Using numerical simulation, we have shown our methods can effectively desynchronise these systems with greater efficacy than both CR and PL stimulation. Most importantly perhaps is that our work sheds light on the importance of the state for DBS strategies. Previous experimental studies have demonstrated the effectiveness of phasic stimulation [17, 19, 20]. Our theories can explain these findings, but also suggest that this approach would be suboptimal in general and that greater knowledge of the state, in particular the local phases and amplitudes, is required to improve efficacy.

The mathematical description of ACD also predicts the utility of closed-loop multi-contact DBS to be largely dependent on the form of the uPRC and in particular on the zeroth harmonic *a*_0_, which is related to whether it is type I or type II. ACD is expected to have the greatest utility (relative to PL stimulation) for type I systems, where |*a*_0_| is large relative to other harmonics. Systems with type I uPRCs are described by a significant number of neuron models and generally fall into the category of Class I excitable [44]. For type II systems, where |*a*_0_| is small relative to other harmonics, stimulation on the basis of local quantities is unlikely to be beneficial. We also show that the dependency of the amplitude response on the local quantities of population *σ* becomes less at increasingly lower local amplitudes *ρ*_*σ*_ but that the effects of stimulation are, in general, explicitly dependent on the state of the system.

### Limitations and future work

The simulations we present provide only a broad understanding of the potential efficacy and efficiency of ACD and there is scope for future work. The formulation of ACD relies on a number of assumptions, one of which concerns the oscillator distribution of each population satisfying the *ansatz* of Ott and Antonsen [32]. This *ansatz* is known to correctly describe the mean-field behaviour for an infinite Kuramoto system but may not necessarily be a good description for systems with different dynamics. The presence of noise in our simulations is a deviation from the systems described by the *ansatz*. Our results therefore provide a useful demonstration of ACD’s efficacy when applied to a more realistic system. It is currently unclear if this efficacy would be maintained for systems with more complex coupling functions. The presence of higher harmonics in the uPRC may also affect the efficacy of our methods. It is known that the presence of higher harmonics in *Z*(*θ*) can lead to clustering of oscillators, which causes a breakdown in the Ott-Antonsen *ansatz* [45]. In addition to this, we assume a Lorentzian distribution for the natural frequencies– how would the efficacy of the method change if we simulated systems with different distributions? Investigating ACD using a more diverse range of systems, particularly those where these assumptions have been relaxed, would be a good way to test the robustness of the method.

Underlying the method of CR is the assumption of a single population of homogeneously coupled oscillators with identical frequencies [8]. This represents a specific case of the more general system we consider in our testing given by Eq (41). By changing the fixed parameters {*k*_offdiag_, *ω*_0_, *γ*}, we can bring our test system into closer alignment with the assumptions of CR, which may lead to greater efficacy for the method. However, this is unlikely to address the dependence of CR on relatively high stimulation intensities to achieve efficacy, which is perhaps its main limitation and is in contrast to ACD. In addition to this, a single population of oscillators with identical frequencies and increased interpopulation coupling *k*_offdiag_ is unlikely to produce oscillation data that is reflective of either tremor or LFP recordings. Increasing *k*_offdiag_ would also require larger and possibly unrealistic stimulation intensities to achieve a desynchronising effect.

We also make a number of assumptions when modelling the electrostatics of the system, most notable of which is that of ‘small populations’. Localising populations of activity in this way allows us to use some elementary results from electrostatics and connect them with what we already know about the way neural populations respond to DBS. However, we recognise that this assumption might be severe for some systems. We assume small populations when deriving both Eq (31) for the ACD closed-loop strategy and (55) for relating local activities to recordings via ICA. As previously mentioned, the assumption becomes more valid as *S* becomes larger, but in practice, resolving the local quantities for these larger systems would then require more contacts. At present, clinically available systems feature at most 8 electrode contacts, but future systems could feature as many as 40 [46]. In addition to this, the assumption of a point source electrode may be adequate for describing stimulation [36], but could be problematic when modelling LFP recordings, where the geometry of the electrode may play a more important role. Another limitation of our model is that it only describes the *instantaneous* effects of stimulation, rather than those over a finite time period. Using this assumption leads to an important simplification for the response (25), which becomes independent of the parameters describing the dynamics. Real stimulation pulses, however, have a finite duration and more complex shapes. Accounting for these in our model would require an integration of the dynamical equations in addition to those for the response, which would inevitably result in greater complexity. Taken altogether, it is unclear at this stage how these assumptions would affect the efficacy of ACD and further simulation work would be required to shed light on this.

Electrodes which record the population activity are also susceptible to recording the stimulation pulses themselves. This manifests in recordings as an artefact, which poses a challenge for closed-loop methods that rely on the real-time measurement of phases and amplitudes. Addressing the effects of stimulation artefacts is beyond the scope of this work, but we expect that significant suppression of the stimulation artefact would be required for ACD to be effective. This suppression may come as a byproduct of using ICA, which has been found by others [47, 48]. Alternatively, by recording through two contacts adjacent to a single stimulating contact, the properties of differential amplifiers can be used to suppress the stimulation artefact [49]. It’s also worth mentioning that we have only considered perturbations to neural populations using electrodes, but in principle, our theories should also be valid for other types of perturbation, such as optogenetic, where light pulses are used to perturb genetically modified neurons [50]. This approach would eliminate the stimulation artefact and could potentially improve the real-world performance of ACD.

Overall, this study represents an important and necessary first step towards implementing our ideas into practice. Beyond this, and on the theoretical side, further steps should include bringing our methods of testing into closer alignment with an experimental paradigm, ultimately treating the system as a ‘virtual patient’, with the only inputs available to ACD being simulated electrode measurements *V*(*t*). A simulated artefact from stimulation may also be included. The various parameters of (26) would then be estimated from these and the state variables obtained using ICA and (54). Introducing uncertainty into the parameters and state will almost certainly affect the efficacy of ACD but understanding the extent of this effect will help us better gauge the potential real world performance and feasibility of the method.
